# The Impact of Environmental and Endogenous Damage on Somatic Mutation Load in Human Skin Fibroblasts

**DOI:** 10.1371/journal.pgen.1006385

**Published:** 2016-10-27

**Authors:** Natalie Saini, Steven A. Roberts, Leszek J. Klimczak, Kin Chan, Sara A. Grimm, Shuangshuang Dai, David C. Fargo, Jayne C. Boyer, William K. Kaufmann, Jack A. Taylor, Eunjung Lee, Isidro Cortes-Ciriano, Peter J. Park, Shepherd H. Schurman, Ewa P. Malc, Piotr A. Mieczkowski, Dmitry A. Gordenin

**Affiliations:** 1 Genome Integrity and Structural Biology Laboratory, National Institute of Environmental Health Sciences, US National Institutes of Health, Research Triangle Park, North Carolina, United States Of America; 2 School of Molecular Biosciences, Washington State University, Pullman, Washington, United States Of America; 3 Integrative Bioinformatics Support Group, National Institute of Environmental Health Sciences, US National Institutes of Health, Research Triangle Park, North Carolina, United States Of America; 4 Department of Environmental Science and Engineering, University of North Carolina, Chapel Hill, North Carolina, United States Of America; 5 Department of Pathology and Laboratory Medicine, University of North Carolina, Chapel Hill, North Carolina, United States Of America; 6 Epidemiology Branch, National Institute of Environmental Health Sciences, US National Institutes of Health, Research Triangle Park, North Carolina, United States Of America; 7 Department of Biomedical Informatics, Harvard Medical School, Boston, Massachusetts, United States Of America; 8 Division of Genetics, Brigham and Women’s Hospital, Boston, Massachusetts, United States Of America; 9 Clinical Research Unit, National Institute of Environmental Health Sciences, US National Institutes of Health, Research Triangle Park, North Carolina, United States Of America; 10 Department of Genetics, Lineberger Comprehensive Cancer Center, University of North Carolina, Chapel Hill, North Carolina, United States Of America; University of Edinburgh, UNITED KINGDOM

## Abstract

Accumulation of somatic changes, due to environmental and endogenous lesions, in the human genome is associated with aging and cancer. Understanding the impacts of these processes on mutagenesis is fundamental to understanding the etiology, and improving the prognosis and prevention of cancers and other genetic diseases. Previous methods relying on either the generation of induced pluripotent stem cells, or sequencing of single-cell genomes were inherently error-prone and did not allow independent validation of the mutations. In the current study we eliminated these potential sources of error by high coverage genome sequencing of single-cell derived clonal fibroblast lineages, obtained after minimal propagation in culture, prepared from skin biopsies of two healthy adult humans. We report here accurate measurement of genome-wide magnitude and spectra of mutations accrued in skin fibroblasts of healthy adult humans. We found that every cell contains at least one chromosomal rearrangement and 600–13,000 base substitutions. The spectra and correlation of base substitutions with epigenomic features resemble many cancers. Moreover, because biopsies were taken from body parts differing by sun exposure, we can delineate the precise contributions of environmental and endogenous factors to the accrual of genetic changes within the same individual. We show here that UV-induced and endogenous DNA damage can have a comparable impact on the somatic mutation loads in skin fibroblasts.

**Trial Registration:**

ClinicalTrials.gov NCT01087307

## Author Summary

Somatic genomes are constantly accumulating changes caused by endogenous lesions, errors in DNA replication and repair, as well as environmental insults. Despite the importance of somatic genome instability in aging and age-related pathologies, including cancers, accurate measurements of mutation loads in healthy cells is still missing. In this study, we developed an experimental approach to accurately determine the somatic genome changes accrued in cell lineages over the lifetime of healthy humans. We show that the amounts and types of mutations in skin cells resemble many cancers, thus indicating that the mechanisms that lead to carcinogenesis are also functional in healthy cells. Moreover, sun-exposed skin cells have a higher mutation load attributable to ultraviolet radiation (UV) unlike cells from hips that were protected by clothing. Our work provides precise measurements of the mutation loads in single cells in human skin. Furthermore our data allowed defining the mutagenic impacts of environmental and endogenous processes within the same individual and led to conclusion that these processes have a comparable impact on the somatic mutation load.

## Introduction

Endogenous DNA lesions and inaccuracies in replication and repair, as well as environmental DNA damage result in a buildup of mutations throughout the human genome over the lifetime of the individual. Studies with transgenic mice have demonstrated that genome instability increases with age and in agreement with tissue-specific proliferation capacities [1, 2]. Also, somatic mutation frequency in human cancers has been shown to increase with the age of the patients at the time of tumor excision [3–5] and various studies using reporter systems in human cells have demonstrated that the increase in mutations with age is dependent on the tissue-type being tested (reviewed in [6]). Central to all models of somatic mutation accumulation with age is the hypothesis that mutation loads are a product of endogenous and environmental mutational processes. However, the impact of these factors on carcinogenesis has only been assessed indirectly. A comparison of mutation data in various types of tumors with the levels of cell proliferation in the affected tissues led to the conclusion that DNA replication-associated mutagenesis in non-cancerous stem cells is the foremost contributor to carcinogenesis [7]. Alternatively, taking into account epidemiological studies and mutation signatures associated with known mutagens, the primary risk factor for cancers has been proposed to be environmental [8].

Buildup of somatic genetic changes during human lifetime was proposed to result in somatic mosaicism, and the existence of such mosaicism, had been recently demonstrated by several groups (reviewed in [9, 10]). This led to the next set of questions about defining spectrum and measuring rates of genome changes resulting in somatic mosaicism. *In vivo*, mutation rates in human somatic cells were previously measured in single-gene reporters or by detecting mutations in a small fraction of the genome (reviewed in [10, 11]). Such estimates are unsuitable for genome-wide extrapolation because they vary considerably (from 10^−9^ to nearly 10^−7^ per nt/cell division [12, 13]) and because rates of changes differ across the genome [14]. In a recent study, mutations characteristic of ultraviolet (UV) radiation in human somatic tissues, normally exposed to UV over lifetime, were analyzed by conducting ultra-deep sequencing of 74 genes in DNA extracted from eyelid biopsies as well as whole genome sequencing of DNA obtained from a mixture of cells within one biopsy [15]. This approach is well suited to detect somatic changes accumulated in a significant fraction of cells either by chance or due to selection. However, when a bulk of cells directly obtained from a biopsy are sequenced, the detection and validation of somatic mutations unique to a single cell or present in a very small fraction of cells in a given tissue, is extremely challenging and precludes precise determination of the scale of mutagenesis over an individual’s life time. While single-cell sequencing analyses have facilitated the estimation of genome-wide mutation load in individual non-cancerous human cells, such assays have unavoidable amplification errors which can significantly obscure mutation calling and the method does not allow independent validation of mutation calls [16]. Sequencing of induced pluripotent stem cell lines could circumvent this difficulty, however, generation of such lines is inherently mutagenic [17]. As such, precise determination of mutation loads in human somatic cells is still missing.

We report here accurate estimates of various types of somatic genome changes accumulated in somatic cell lineages, across the entire genome. Moreover, our experimental design enabled us to compare mutagenesis contributed by endogenous and environmental factors within the same individuals, leading to the conclusion that the mutagenic impacts of these processes are comparable.

## Results

### Experimental design

Normal fibroblasts reside in the connective tissues and are responsible for generating extra cellular matrix. Oncogenic transformation of cells within the connective tissue leads to fibrosarcomas. Studies in mice have demonstrated that histologically normal epithelial cells can be induced to form tumors by generation of oncogenic mutations in the stromal fibroblasts [18, 19]. Fibroblasts isolated from the stroma of various human cancers including melanoma also have higher proliferation rates and capacity. Thus, emerging evidence has demonstrated that besides a supportive role in cancer initiation and progression, cancer-associated fibroblasts can also be drivers of carcinogenesis [20], which makes it pertinent to study the acquisition of somatic mutations in these cells in healthy individuals over time. Importantly, skin fibroblasts are readily accessible from small punch biopsies and possess proliferative potential enabling clones derived from single cells in primary culture to grow to sufficient cell density for DNA library preparation and next-generation DNA sequencing. This method allows for accurate estimation of the mutation loads in the progenitor cell that gave rise to the clonal human cell lines, and permits systematic verification of the somatic changes.

**Fig 1:**
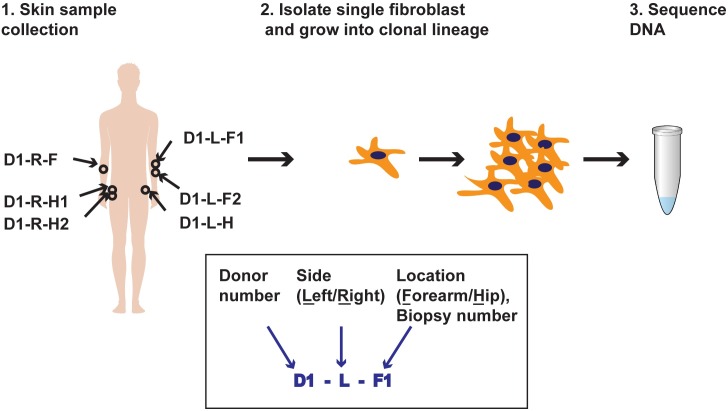
Schematic for isolation and sequencing of single-cell fibroblast clonal lineages. Example of Donor 1 is shown. Boxed insert illustrates the design of the clone IDs. Biopsy number is indicated if adjacent biopsies were taken from the same site.

We sequenced the genomes of 10 clonal fibroblast cell lineages, generated by limited propagation of cells obtained from skin punch biopsies. The biopsies were obtained from the left and right forearms and hips of two healthy donors (ages 58 and 62), where significant fraction of cells retained the capability to grow in culture (Fig 1 and S1 Fig). For Donor 1, we obtained two additional biopsies adjacent to the initial positions at the right hip and left forearm (Fig 1). We reasoned that the forearms would have had greater exposure to sunlight than hips, which would allow estimation of the impact of this environmental factor on the accumulation of genome changes as compared with unexposed sites within the same donor. Studies on reconstructed skin have determined that unlike UVB rays that are only capable of penetrating the epidermis, UVA rays can reach the dermal layer [21]. Also, fibroblasts in sunlight-exposed skin are subject to UVA-induced damage producing wrinkles, solar elastosis [22] and likely several forms of mutagenic DNA damage. Thus, we expect to detect an excess of UV-induced mutations in the forearms of both donors.

Single fibroblasts were isolated from each biopsy and expanded clonally in culture for 5–6 additional passages (4–6 weeks) to obtain approximately 10^6^ cells. Whole genome sequencing was performed to get 40-60X coverage for each sample. Whole blood was also obtained from each donor and DNA was extracted and sequenced to a similar depth. Over 91% of the genomes of each clone had a minimum coverage of 10 reads (S1 Table). All changes present in the clones and absent from the corresponding sequenced blood DNA, which is made up of a heterogeneous cell population, and whose mutation calls and other genome changes likely represent the germline genotype of a donor, were deemed somatic in origin and unique to the fibroblast clones.

### Detecting retrotransposition events in the clones

We searched for somatic retrotransposition events in the clones similar to those shown for cancer genomes of epithelial origins [23, 24]. However, in the 10 genomes from normal skin fibroblasts, we detected only two LI insertion candidates, one of which showed the signatures of a *bona fide* retrotransposition by target primed reverse transcription—14 bp target site duplication and poly-A tail. But, the clonality of the events was estimated to be 2–12% (S2 Fig and S2 Table), suggesting that they were acquired during the clonal expansion and were not present in the progenitor cell that gave rise to the clone. Our analysis supports the view that somatic retrotransposition is inhibited to a greater extent in fibroblasts than in germ cells [25].

### Genome-wide identification of somatic structural changes

Large scale somatic copy number variations (CNVs) have been previously implicated in neurological diseases, autoimmune diseases, heart diseases and cancers [26]. Through analysis of sequencing read depth by VarScan2 and correction for GC content [27, 28], we detected a total of 57 clonal somatic deletions in the genomes of skin fibroblasts resulting in a ploidy of 1n, and 13 amplification events with a ploidy of 3n. Each clone was found to contain more than one (ranging 2 to 22) somatic copy number variation (CNV). Most CNVs were focal in nature, however, we found 5 CNVs that span over the entire chromosomal arm in 3 out of the 10 clones (S3 Table).

**Fig 2:**
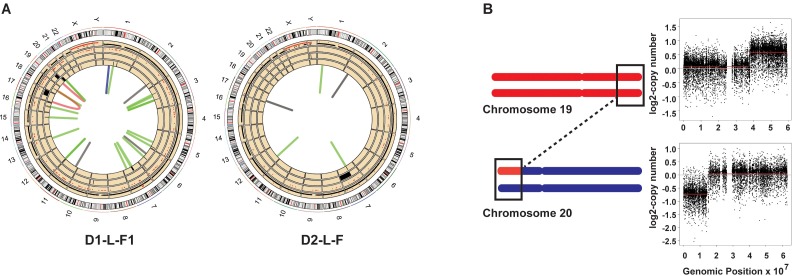
Structural changes detected in skin fibroblast clones D1-L-F1 and D2-L-F. (A) All genome changes detected in D1-L-F1 and D2-L-F clones. The tracks numbered from innermost are as follows: 1—structural changes. Green = deletions, black = duplications, blue = inversions and red = translocations. 2—deletions as detected by read-depth analyses. 3—amplifications as detected by read-depth analyses. 4—LOH events. 5—somatic SNVs, black dots are heterozygous SNVs and red dots are homozygous SNVs. (B) Schematic describing the chr19, chr20 translocation in D1-L-F1. Black rectangles depict region wherein translocation event was detected with a concomitant change in copy number.

We also detected 57 somatic structural changes with formation of novel junctions based on analysis of split and discordant paired-end reads by DELLY [29]. Only calls with >30% of the reads supporting the novel junctions were deemed clonal. This allows us to eliminate sub-clonal structural variations that may have occurred in the clones during propagation in culture. Many rearrangements were further supported by the independently called CNVs mentioned above, and by the presence of loss of heterozygosity (LOH) tracts in the regions with heterozygous deletions (Fig 2A, S3 Fig, S4 Fig, S3 Table, S4 Table and S5 Table). LOH events are called by VarScan2 as somatic, when a base pair different from the reference genome GRCh37, heterozygous in blood DNA, turns homozygous in the clone from the same donor. We validated 34 somatic structural changes by PCR and Sanger sequencing the breakpoints (S5 Fig and S4 Table).

Interestingly, several structural rearrangements were present in the vicinity of known fragile sites (data taken from [30]). For example, the chr20 left arm has a 15 Mb terminal deletion, and the 21 Mb terminal region of the right arm of chr19 is amplified to 3 copies. We also detected a translocation between chromosome 20 and chromosome 19 in the regions with copy number changes. We posit that such a rearrangement might occur from double strand break in chr20 followed by break-induced replication over the right arm of chr19 (Fig 2B and S4 Table). The region comprising the breakpoint in chr20 is a known rare fragile site and rearrangements involving this region have been implicated in the pathology of Alagille syndrome and in colorectal cancers [31–33]. Also, 3 of the 4 clones from donor2 and 1 clone (left forearm) from donor1 carry different heterozygous deletions in the NRXN1 locus of chr7 (S4 Table). This region is also a known hotspot for structural variations and deletions of this locus have been implicated in the autism spectrum disorders [34]. This locus is enriched in small inverted repeats that have been shown to lead to replication fork stalling and gross chromosomal rearrangements [35–37]. These rearrangements implicate difficulties in DNA replication and breakage at fragile sites as a frequent mechanism to generate CNVs in somatic cells. Overall, large scale structural changes in the clones leading to copy changes were 4 to 10—fold lower than the median levels of CNVs detected in cancer genomes [38] and did not indicate any impact of sun exposure.

### Genome-wide catalogs of somatic base-substitutions

**Fig 3:**
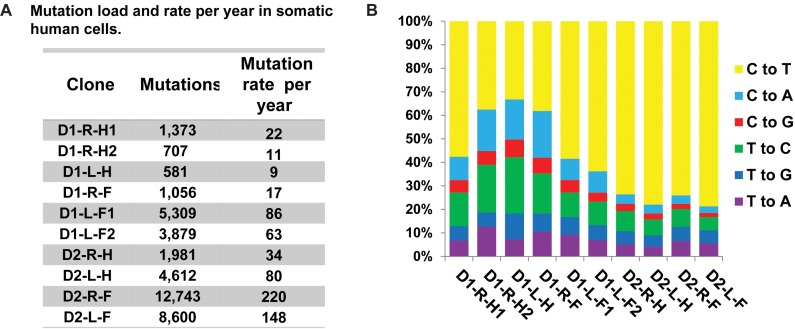
Somatic mutation load and spectra in the fibroblast clones. (A) The number of somatic mutations detected in each clone and the rate of accumulation of mutations per year are provided. (B) The spectra of base changes in the clones. For each base change the reverse complements are also included.

To obtain accurate estimates of the somatic single nucleotide variations (SNVs) in the progenitor cell that gave rise to the clonal lineages, we obtained consensus somatic mutation calls from three independent callers: the haplotype caller from GATK [39], VarScan2 somatic mutation caller [27], and MuTect [40]. Somatic base-substitutions in the clones represented the largest number of genetic changes, from 581 to 12,743 (Fig 3A). SNVs were considered clonal and somatic if they were absent in the blood DNA and had allele frequencies between 45% and 55% or greater than 90% (S6 Fig, S6 Table and S7 Table). These filtering criteria allowed us to exclude mutations that are coming from germline as well as those that may have arisen in the first cell division in culture. 102 of the SNVs identified as somatic and clonal were PCR amplified and Sanger sequenced for further validation. Of these, we were able to get amplification and sequencing results for 87 loci containing SNVs, and all were validated as true somatic events (S6 Table). We uncovered only one shared mutation between clones isolated from Donor 1, from the left forearm, and two mutations common in clones isolated from the forearms of Donor 2 (S7 Fig). We propose that the dearth of mutations common to clones from the same subject is due to the high cell turnover rates in the dermis. Indeed, the area of human skin increases only 6-fold in postnatal development to adults [41]. If turnover rate is uniform across the body only six fibroblasts of the adult dermis would share a common ancestor from the neonatal stage.

Exome sequencing from bulk fibroblast cultures to 100X – 200X coverage, revealed the presence of a large number of somatic mutations identical to mutation calls in clones (264/387) (S6 Table). 90% or more of the SNVs detected in the exomes of each sample have frequencies ≥ 5%, while mutations with as little as 2% frequencies were also called (S8 Fig and S8 Table). All samples except D2-L-F contain 1 peak with the majority of SNVs having allele frequencies between 5–15%. In the D2-L-F bulk sample, the SNVs are distributed between 2 peaks with allele frequencies between 5–15% and 25–50%. This distribution of allele frequencies implies that there is 1 major clonal lineage present in this population thus contributing to high allele frequencies of the SNVs detected, and a smaller population comprised of multiple clonal lineages contributing to mutations with frequencies in the range of 5 and 15%. The distribution of allele frequencies can be explained if there are at least 10 clones present within each biopsy. This hypothesis is corroborated by the observation that the numbers of somatic mutations detected in the exomes of bulk cells are comparable to the expected number of mutations if there were at least 10 clones similar to those isolated from the same biopsies (S9 Table). These data point towards the highly heterogeneous nature of skin. Altogether, deep exome sequencing of the source bulk cultures further confirmed that the SNVs detected in the clones were present in the skin biopsy and were not an artifact of growing the cells in culture.

### Human somatic mutation rates

Based on the shortening of telomeres in the fibroblast clones we estimated that the cells underwent a minimum of 60 to 70 post-natal cell divisions in the body prior to biopsy and clone formation (see Methods). Therefore, the maximal estimates of somatic mutation rates in clones accounting for only the postnatal divisions are ~1x10^-9^ to 3x10^-8^/nucleotide/cell division. Assuming that genetic changes occurred at a steady rate in the somatic cells, we can further calculate the rate of mutation accumulation in skin fibroblasts to be 9–220 mutations/genome/year (Fig 3A and S10 Table). This is close to the estimates of age-dependent accumulation of certain mutation types in cancer genomes [5].

### Mutation signatures in the clones

**Fig 4:**
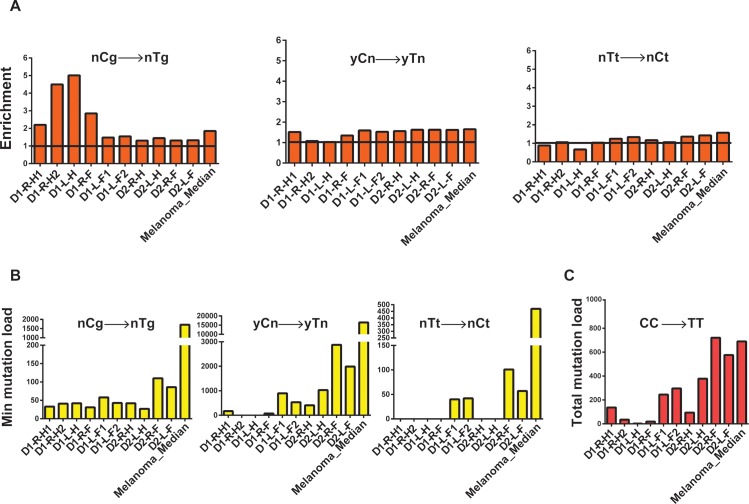
Analysis of mechanistic knowledge-based mutation signatures in the genomes of skin fibroblasts. Similar analysis for the whole-genome sequenced melanoma (SKCM) cohort (dataset from [46]) is provided for comparison. (A) Fold enrichment of nCg →nTg and UV-signature mutations (yCn→yTn, nTt→nCt; y is either C or T, n is either A, T, G or C, in the trinucleotide context the mutated base is in capital). The black horizontal line denotes the level of no enrichment. (B) The minimum estimates of signature-specific mutation loads for each clone. For the melanoma cohort, the median of the minimum estimated mutation loads for each signature per genome in is shown. (C) Total CC→TT counts of tandem dinucleotide changes in each clone and the median of the total CC→TT counts per genome in the melanoma cohort.

The overall mutation loads in fibroblasts obtained from the forearms were higher than the fibroblasts isolated from hips of the same donors suggesting the involvement of UV in the origin of mutations in the forearms. In our samples, the most prevalent base change was C→T (Fig 3B), which is the prevailing change in several mutagenic pathways [42]. We therefore explored mutation spectra in clones by applying statistical analysis based on prior mechanistic knowledge using a similar approach as previously implemented for APOBEC cytidine deaminase-induced mutagenesis in human cancers (outlined in [42–44] and Methods), which provides sufficient power to detect mutagenic patterns in individual samples. In brief, we determined if counts of mutations at a given motif, consistent with a specific mutagenic mechanism, are statistically enriched as compared to mutation counts expected by random mutagenesis. For samples with statistically significant enrichment we then calculate the minimum estimates of mutation loads attributable to the mutagenic mechanism. One known pathway of C→T mutagenesis is initiated by deamination of methylated cytosines in CpG dinucleotides [42]. Mutations at methylated CpG dinucleotides leading to C→T changes have been shown to increase in a “clock-like” manner with age [5, 45]. Indeed, we found an enrichment of C→T mutations at nCg motif (shown in trinucleotide format, mutated nucleotide capitalized, n is any nucleotide) in all clones analyzed (Fig 4A and 4B, and S11 Table). However, this load represented no more than 5% to 40% of all C→T changes, indicating that other mutational mechanisms are also functional in the clones.

UV-A exposure leads to the formation of mutagenic cyclobutane pyrimidine dimers and pyrimidine (6–4) pyrimidone photoproducts [47, 48]. Deamination of cytosines forms uracil leading to C→T changes in the genome and this rate is 10^6^-fold higher for cytosines within cyclobutane pyrimidine dimers [49] To evaluate the contribution of UV-induced mutagenesis in the single cell-derived fibroblast clones we examined C→T changes in the 3’C present in TpC or CpC contexts, and tandem CpC→TpT dinucleotide changes [15, 48, 50]. We observed a higher incidence of these substitutions in most forearm samples as compared to hips in the same donor (Fig 4A–4C and S11 Table).

Analyses of trinucleotide motif-specific mutation signatures enable us to detect apparent contribution of UV-mutagenesis even when there is an overlap with a mutation signature commonly associated with endogenous processes. Mutations at CpG motifs leading to C→T changes have been attributed to age-dependent mutation accumulation due to intrinsic deamination of methylated cytosines. However, CpG motifs present in the yCg trinucleotide contexts have also been shown to be efficiently mutated by UV because of higher efficiency formation of pyrimidine dimers, which include methylated cytosine [51, 52]. In agreement with such a preferred dimer formation, enrichment and minimum estimates of mutation loads attributable to the yCg→yTg mutation signature were higher than for rCg→yTg in most samples, suggesting that a large fraction of yCg→yTg mutation are due to UV-induced DNA damage rather than to spontaneous deamination of 5-methyl cytosine in 5me-CpG (nCg→nTg) mutation load (S9A Fig). Alternatively, this could be caused by, not yet identified, influence of the nucleotide 5’ to meCpG on the rate of spontaneous deamination of meC resulting in C→T mutations.

Since, we clearly detected a significant contribution of UV-induced mutagenesis in C→T changes in the clones from the forearms; we further analyzed the role of UV in mutagenizing A:T base pairs as well. UV-induced dimers can be also formed between adjacent thymines. Consistently, in the clones derived from forearms we see increased levels of TT→CT base substitutions in the forearms as compared to hip samples from the same donor (Fig 4A and 4B and S11 Table).

In order to support the accuracy of our mechanistic knowledge based signature analysis for UV-induced mutations, we further analyzed published whole-genome mutation catalogues of melanomas [46]. Melanomas are known to have a large number of UV-induced mutations and whole genome mutation calls were available for this cancer type. We clearly see high incidence of all three kinds of UV-signature mutations in these genomes (Fig 4A–4C and S11 Table). Similar UV-signature mutations have also been found in cutaneous squamous cell and basal cell carcinomas, where only exomes were sequenced ([15] and references therein). *In vitro* and in yeast, bypass of UV-induced photoproducts at TT tandem bases by the translesion polymerase Polƞ, predominantly leads to a change in the 3’T causing a TT→TC base substitution[53, 54]. However, in healthy skin cells, and in melanoma samples we detect an enrichment of mutations in the 5’T (Fig 4A and 4B and S11 Table), indicating differences in the mechanism underlying UV-induced lesion bypass in humans and model systems. One explanation for this bias towards mutations in the 5’T could be that there is an overrepresentation of pyrimidines in the -1 position in the nTt motif in our dataset. Formation of pyrimidine dimers at YT dinucleotides, and bypass could lead to T→C changes, thus confounding our analyses centered on the nTt motif. Therefore, we determined enrichment and minimum mutation loads for the non-overlapping parts of nTt motif—yTt and rTt (y is a pyrimidine, r is a purine). Interestingly, we clearly detect higher enrichment and mutations leading to rTt→rCt changes than yTt→yCt changes (S9B Fig, S11 Table), thus supporting our conclusion that in normal human cells and in melanomas, the 5’T in a thymine dimer is preferentially mutated and leads to T→C changes.

### Mutation density correlates with replication timing and chromatin status

**Fig 5:**
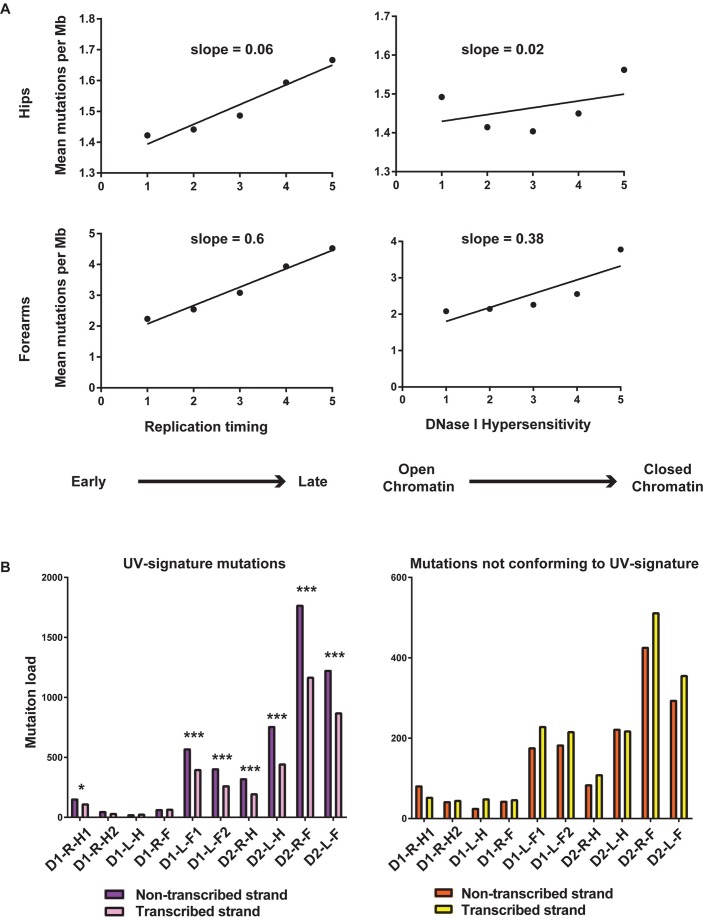
Somatic mutation loads vary with replication timing and transcription status. (A) SNVs from both, hips and forearms are enriched in late replicating genomic regions and heterochromatic regions of the genome. Values on the horizontal axis were obtained by averaging values of the genomic feature into 5 equal bins. (B) UV-attributable mutations demonstrate a strand bias for the non-transcribed strand. * denotes p-value after false discovery rate (FDR) correction for multiple hypothesis testing < 0.05, *** denotes p-value after FDR correction < .01 (p-values are in S13 Table). There is no preference for non-transcribed strand for mutations in C and T which do not conform to any UV-signature motif.

Mutation rates in cancers as well as in germline vary between regions of the genome that replicate early or late, and that have open or closed chromatin [14, 55, 56]. This tendency for increased mutations in repressed chromatin was also detected in whole-genome sequenced DNA from a single skin biopsy obtained from eyelids of healthy individuals [15]. To assess the dependence of somatic mutagenesis on these epigenomic features we used Repli-seq data for BJ cells (derived from normal foreskin fibroblasts) from the ENCODE project [57] and DNase I hypersensitivity data from NHDF-Ad cells (adult dermal fibroblasts). These cell types were selected as they were the closest in origin to the fibroblast clones analyzed in our study. Like cancer genomes, where late replicating and heterochromatin-rich regions have more mutations than early replicating euchromatic regions [55, 56], SNV density in the fibroblast clones, correlated with replication timing and the chromatin status. This bias was more pronounced in forearm samples than in the hips (Fig 5A, S10 Fig, S11 Fig and S12 Table), and was also observed for UV-induced mutations (S12 Fig and S12 Table).

### Transcription-associated bias for SNV density

UV-signature mutations present within transcribed regions of the genome were further classified based on whether the mutations occurred in the transcribed or non-transcribed strand. In four out of five forearm samples and in three out of five hip samples we observed more of these mutations in the non-transcribed strands (Fig 5B and S13 Table), which is in accordance with the activity of transcription-coupled repair [15, 45, 58]. Analysis of mutations of pyrimidines that did not conform to any of UV-mutagenesis signatures did not demonstrate bias towards the non-transcribed strand.

## Discussion

Determination of somatic mutation loads accumulated in healthy individuals has been difficult due to the underlying inaccuracies of the methods used to obtain mutation spectra in single-cells. In this study, through establishment of single-cell clonal lineages with no re-programming and with minimal propagation or manipulation post extraction from the human body, we have determined the different types and extent of somatic genome changes that accumulate in skin fibroblasts in two healthy individuals. Our approach allows us to accurately identify changes that were present in a single cell in skin, and to further validate these changes independently.

We found that each somatic cell carries at least one somatic structural variation leading to change in copy number. Previously, genome sequencing of skin-fibroblast-derived cell lines led to the prediction that 30% of these cells carry large scale CNVs [59]. Also, single cell analysis of neurons from healthy individuals and a patient with hemimegalencephaly demonstrated the presence of megabase scale somatic CNVs [60]. Our analyses reveals that somatic CNVs are commonly present in the human genome and are likely stemming from double strand break formation during DNA replication over fragile sites in the genome.

We demonstrate that the mutation load in healthy cells (ranging from 581 to 12,743 mutations, Fig 3A) can be comparable to the mutation loads in some cancer genomes (median ~6000 mutations/cancer genome [61]) and the spectra as well as the correlation of base substitution density with epigenomic features resemble many cancers. Apparent variability of mutation loads between the donors precludes generalizing our mutation rate measurements to a population-scale, nonetheless our study provides accurate estimates of the impacts of both UV and endogenous mutational processes on the mutation loads in skin fibroblasts of healthy human individuals. Since, the fibroblasts in the dermis of the hips would have had no or much lower UV exposure as compared to forearms, we inferred that mutations not attributable to UV were most likely representative of endogenous mutagenic processes in these samples. On the contrary, mutations in forearms would carry a greater fraction of UV-induced mutations. We note that some of the UV-like mutations might be caused by other non-UV processes, and/or UV-induced damage may culminate into, hitherto unknown, non-UV-signature mutations. Therefore, the conservative approach would be to count endogenous mutations in hip cells as the total mutation loads minus minimum estimate of mutations due to UV (referred to as Endogenous (cons.) in Table 1), and the minimum estimates UV-induced mutation rates from the forearms as an assessment of mutagenesis by this environmental factor (referred to as UV-induced (min.) in Table 1). Comparison of these two categories of mutations suggests that UV-induced and non-UV mutation rates are highly similar in skin fibroblasts (Table 1). We also used an alternative approach for estimating mutagenesis by endogenous damage taking all mutations from the hips of a donor as a maximum estimate of endogenous mutagenesis (referred to as Endogenous (max.) in Table 1) to be also compared with UV-induced (min.) value for the same donor. This approach also leads to the same conclusion that the rate of mutagenesis by environmental DNA damage (UV) is comparable to the endogenous mutation rates in human skin fibroblasts. These data indicate that the mutation loads can be modulated to a similar extent by environmental and endogenous DNA damage in somatic cells.

**Table 1:** Estimates of rates of accumulation of endogenous and UV-induced mutations per year.

Site	UV-induced mutation rate per year^a^	Endogenous mutation rate per year^b^	Total mutation rate per year^c^	Source of damage–Approach 1^d^	Average rate of mutations by source–Approach 1	Source of damage–Approach 2^e^	Average rate of mutations by source–Approach 2
D1-R-H1	5	13	22	Endogenous (cons.)	9.7	Endogenous (max.)	14.0
D1-R-H2	2	9	11				
D1-L-H	2	7	9				
D1-R-F	3	12	17	UV-induced (min.)	10.3	UV-induced (min.)	10.3
D1-L-F1	17	51	86				
D1-L-F2	11	36	63				
D2-R-H	9	24	34	Endogenous (cons.)	32.5	Endogenous (max.)	57.0
D2-L-H	20	41	80				
D2-R-F	54	127	220	UV-induced (min.)	46.0	UV-induced (min.)	46.0
D2-L-F	38	84	148				

a UV-induced mutation rates are calculated as the sum of minimum mutation loads by the yCn→yTn, nTt→nCt and CC→TT mutation signatures, divided by the age of the donors.

b Endogenous mutation rate per year is calculated by subtracting minimum mutation loads attributable to UV from the total mutation loads in the clones and dividing by the age of the donors.

c Total mutation rate per year is calculated by dividing the total mutation load by the age of the donors.

d In Approach 1, average Endogenous (cons.) mutation is calculated as the average of the “Endogenous mutation rate per year” for the hip samples of each donor. Average UV mutation rates in forearms are the average of the “UV-induced mutation rate per year” for the forearm samples of each donor.

e In Approach 2, average Endogenous (max.) mutation rate is calculated as the average of the “Total mutation rate per year” for the hip samples of each donor and compared to the average UV mutation rates in forearms of the same donor.

The mutation load per fibroblast was at least 600 substitutions (Fig 3A). In an average human body there are approximately 10^12^ dermal fibroblasts [62], thus the skin as a whole could carry ~10^14^ SNVs. However, based on the observed allele fractions of somatic mutations revealed by deep exome sequencing of bulk cultured cell populations from 8 biopsies, a significant fraction of these SNVs would be the same, and thus belong to the same clonal lineage (S8 Fig, S8 Table and S9 Table). Such a clonality of mutations could be due to a combination of random cell death, mutation specificity, and selection, which would result in high incidence of certain somatic changes. Recent study based on analysis of TCGA sequence data for normal tissues adjacent to excised tumors did not find evidence of strong purifying selection against new potentially deleterious mutations [63]. In fact, another study involving deep sequencing of genomic DNA obtained from non-cancerous human skin indicated that there is even some level of selection for changes in certain genes implicated in carcinogenesis [15]. Interestingly, 23 out of 387 somatic mutations in exons were also present in the COSMIC database (S6 Table). We propose that similar to significantly mutated genes in cancers [61], there are genes mutated in normal skin cells above random frequency expectations. Our work provides a platform for testing this hypothesis by systematic exploration of somatic mosaicism in humans.

The somatic mutation loads in single-cell lineages provide information about an individual’s lifetime history of mutagenic exposure and the impact of intrinsic factors on mutagenesis. Expanding this study to more cell types and a larger population would further refine estimates of the rates of somatic changes in human genomes. Understanding the contributions of environmental and endogenous mutagenic processes to somatic mutation loads is fundamental in developing preventive strategies, and in building better diagnostics and therapeutics to combat cancers and other genetic diseases.

## Materials and Methods

### Sources of cells

All participants gave written informed consent for tissue donation through the Sample Collection Registry for Quality Control of Biological and Environmental Specimens and Assay Development and Testing protocol (registered with ClinicalTrials.gov, number NCT01087307, and approved by the NIH Institutional Review Board at the National Institute of Environmental Health Sciences (NIEHS), protocol # 10-E-0063). We obtained 4 mm diameter skin punch biopsies from the left and right lateral forearms and hips and whole blood from two Caucasian male donors, ages 58 and 62 (Fig 1). Areas for biopsy were identified as free of moles, hair and previous scars. Lateral forearm was away from area that would be covered by a watch. Hip area was under area that would be covered by shorts but away from groin. The skin was sectioned into approximately 6 parts and the sections were allowed to adhere to a cell culture dish. Dulbecco’s modified eagle’s medium (Gibco 11965118) supplemented with 1X non-essential amino acids (ThermoFisher 11140–050), 10% Cosmic Calf Serum (Hyclone SH30087), 10% AmnioMax C-100 supplement (Gibco 12-556-015) and 100μg/ml primocin (Invitrogen ant-pm-1) and the cultures were incubated at 37°C in a 5% carbon dioxide containing incubator. Fibroblasts were selected for their ability to grow well in culture with high cloning efficiency (4–5%). They were identified based on their elongated shape and ability to grow adhered to the tissue culture dishes in the presence of serum-containing media (S1 Fig). It took approximately 3–4 weeks for confluent fibroblast growth from the tissue fragments. The fibroblasts were harvested, diluted and plated to obtain single-cell derived clones. The clones were expanded in culture for, 5 to 6 additional passages (4–6 weeks), to obtain 10^6^ cells and genomic DNA was extracted (DNeasy Blood and Tissue kit, Qiagen). Venous blood for DNA isolation was collected in five 8.5 ml PAXgene blood DNA tubes (PreAnalytiX/Qiagen: Valencia, CA).

### Whole genome/exome sequencing

We used the KAPA Hyper (KAPA Biosystems) kit for whole genome sequencing (WGS) library preparation. Size selection of constructed libraries was performed using Pippin prep (SAGE Science). DNA libraries were used to obtain Paired-End 100 (Rapid V2 chemistry) or 125 bp (V4 chemistry) reads from HiSeq 2500 sequencer (Illumina). Each DNA library was sequenced on 4 lanes to get an average coverage of 40X to 60X for whole genomes, and 100X to 200X for whole exomes (WES) (S1 Table). For whole exome (37 Mb target size) sequencing, Nextera Rapid Capture Exome Kit (Illumina) was used to prepare the library prior to sequencing. The raw FastQ files were filtered to keep only read pairs with an average base quality score of 20 or more and were then aligned to the reference human genome (GRCh37) using BWA-MEM-0.7.10 [64] using the default parameters and the resulting BAM files were merged. Duplicate reads were removed from the resulting BAM files using MarkDuplicates from Picard Tools. The BAM files were processed according to the Genome Analysis Toolkit (GATK) best practices pipeline [39, 65]. The length of the genome with coverage greater than or equal to 10X was estimated using the BedTools genomeCoverageBed command [66] (S1 Table). The final BAM files and complete lists of somatic mutation calls in WGS and WES samples have been deposited into dbGAP approved study under accession number phs001182.v1.p1.

### Detection of somatic retrotransposition and clonality estimation

The published method, scTea [67] was applied to detect insertions of retrotransposons (L1, Alu and SVA) in ten fibroblast clones. At least 5 supporting read pairs and the score higher than 0.6 were required. The germline non-reference insertions were filtered out using the blood genome from the same donor. 13 raw insertion candidates were manually inspected using the Integrative Genome Viewer, and two candidates remained as a final call set (S2 Table). The clonality of each event was estimated by calculating the ratio of discordant reads pairs supporting the insertion (i.e., repeat-anchored mate read (RAM) counts) to the sum of the RAM counts and the number of concordant read pairs across the insertion breakpoint (i.e., those supporting the non-insertion allele). The top high confidence insertion candidate found in the clone D1-L-H (L1#1) resides in a genomic region with large tandem duplication in the germline genome thus having an elevated sequencing depth with poor read mappability whereas the other candidate found in the D2-R-F clone (L1#2) resides in a genomic region with the expected depth and good mappability. To more accurately estimate the clonality of the event like L1#1, we adjusted the number of concordant read pairs by the relative sequencing depth to the genomic average depth and recalculated the allele frequency with an assumption that the insertion allele exists in only one of the many duplicated genomic regions. This copy number adjusted clonality provides us an upper bound of the event clonality (S2 Fig).

### Annotating copy number variations and structural variations

Large-scale clonal copy number changes in the clones were detected using the VarScan2 copy number tool on pileup files generated using Samtools mpileup command [27, 68]. This tool detects changes in coverage in the DNA samples from the clones as compared to their corresponding blood sample. Circular binary segmentation was applied to the output using the DNAcopy package in R [28, 69, 70] (S3 Fig, S4 Fig and S3 Table).

Somatic structural changes in the form of large deletions, inversions, duplications and translocations were detected using the software DELLY [29] using the default settings (S3 Fig, S4 Fig and S4 Table). Alignments of blood DNA reads were used as the “matched normal” to detect clone-specific somatic events. Centromeric and telomeric regions were excluded from the analyses. For each sample a structural variation event was called if > 30% of the reads in the region supported the call and there were 0 reads supporting the event in the corresponding blood sample (S4 Table). The structural variations were further validated by PCR as described below and any variants detected in blood DNA were discarded (S5 Fig).

### Somatic mutation calling and annotation

Single nucleotide variations (SNVs) for WGS samples were analyzed using three mutation calling tools, the haplotype caller from GATK [39], VarScan2 somatic mutation caller from Washington University, St. Louis [27], and MuTect from the Broad Institute of Harvard and MIT [40]. SNVs were called somatic if they were present in the clone and were not detected in the blood of the same donor. VarScan2 and MuTect were run using the alignment files from whole blood from a donor as the “normal” for each clone isolated from this donor. The SNV calls were limited to regions with a minimum of 10X coverage with at least 3 reads supporting the mutation call. The list of SNVs generated were filtered to remove calls that match known dbSNPs (version 138) and calls that fall within known simple repeat regions (UCSC Genome Browser, hg19 build). The consensus between the 3 callers was taken and filtered further for allele frequency to provide the final mutation calls. Calls with an allele frequency between 45% and 55% were denoted as heterozygous mutations, and those with an allele frequency > 90% were denoted as homozygous mutations. Homozygous SNVs may arise in the genome as mutations in genomic regions with copy number = 1 (e.g. X chromosome or autosome region heterozygous for a deletion)or by loss of heterozygosity following a mutation in the autosomes. Mutations with allele frequencies not falling within these criteria were discarded. Since, the samples sequenced were clonal in nature, 61% to 73% of the SNVs called by the three mutation callers were contained within these allele frequencies (S6 Fig, S6 Table and S7 Table).

VarScan2 was also used to annotate loss of heterozygosity (LOH) events in the clones as compared to their respective blood samples. The LOH events in the clones were expected to have an allele frequency > 90% and between 45% and 55% in the corresponding blood samples (S5 Table).

To detect shared SNVs between 2 or more samples, the SNV calls by MuTect were compared for all samples from the 2 donors, respectively. Analyses of allele frequencies for mutation calls by all 3 callers demonstrated that MuTect detects the highest fraction of somatic SNVs. The resulting SNVs were filtered by allele frequency such that calls between 45% and 55% or alleles with frequency > 90% in either of the shared samples were further validated as described below. Only 1 SNV was confirmed to be present in both clones from the left forearm in donor2, and 2 SNVs were validated to be identical between the clones from the left and right forearms of Donor2 (S7 Fig).

Mutations were annotated for their presence within exons and for their functional consequences and potential roles in cancers, using the software Annovar [71] and CRAVAT [72] (S6 Table).

### Exome mutation calling

The bulk fibroblasts that outgrew from the initial biopsies from all 4 sites (forearms and hips) for the 2 donors were used for deep sequencing of the exome with approximately 100-200X coverage (S1 Table) and aligned to the human genome as described above. MuTect was used to call somatic SNVs in these samples. A large fraction (264/387) of the SNVs from the bulk cells were found to be identical to the SNVs detected by the three mutations callers in exomes of the respective clones (S6 Table), thus further corroborating the SNV calls in the clones.

The bulk cells derived from the biopsy from the left forearm of donor 2 showed a bimodal distribution of variant allele frequencies with the 2 peaks at ~5–10% and ~35–45%. 79 of the 83 SNVs present in the exons of D2-L-F (left forearm, donor 2) were present in the bulk sample for the left forearm. 49 of these SNVs had allele frequencies ≥ 40% (S8 Fig, S6 Table and S8 Table). These observations indicate that the biopsy segment for the left forearm for donor 2 primarily contained one clonal lineage from which the clone D2-L-F was derived. In the 7 other bulk samples, the majority of the SNVs called were between 5 and 15% (S8 Fig and S8 Table). These allele frequencies can be explained if each biopsy segment contained a minimum of 10 clonal lineages. Based on this, we estimated the minimum expected exonic mutation loads in a given biopsy segment by multiplying the number of mutations detected in the exome of the clones derived from the bulk cells by 10 (S9 Table).

### Estimation of mutation rate per cell division and per genome per year

The average length of the telomeric repeats was estimated using TelSeq [73]. In newborns, the average length of the telomere restriction fragments (TRFs) has been estimated to be approximately 11 kb [74, 75]. TRFs have been shown to contain 1 kb DNA between the restriction site and telomeric repeats. In order to compare the telomere lengths in our samples to newborns, we therefore added 1 kb to the lengths estimated by TelSeq. Fibroblasts have been shown to lose 50–100 bp of the telomeres every generation [75]. Since, each clone rose from a single cell with at least 20 cell generations, we can assess that telomeres in the progenitor cell were 2 kb longer. This implies that the fibroblast cells that gave rise to the clones have 4–5 kb TRFs. Based on this we can calculate that the cells underwent a minimum of 60 to 70 divisions since the individuals were born. Since the diploid human genome is 6 x 10^9^ nucleotides; we calculate the mutation rate per nucleotide in the genome per cell division as:

$${Rate}_{per\;nt,per\;cell\;division}=\frac{Mutation\;load}{Cell\;divisions\times diploid\;genome\;(nt)}$$

Because the number of prenatal cell divisions is unknown, this calculation represents the maximum estimate of the rate. Therefore, we also calculated mutation rate per genome per year as another characteristics of mutation accumulation (S10 Table).

### Validation of somatic mutation calls, LOH events and structural variants

Primers to amplify regions containing somatic mutations and LOH events were designed using PrimerBlast such that approximately 500 to 1500 bp region spanning the mutated base would be amplified [76]. 6 to 10 mutations in the exons of each clone were chosen for amplification and Sanger sequencing. The corresponding blood DNA was also analyzed to ascertain whether the mutations annotated as somatic were present in the blood (germline). 102 SNVs with potential functional consequences were chosen for validation, of these, 87 SNVs were validated as somatic. We were unable to PCR amplify and get high quality Sanger sequencing results for the remaining SNVs due to difficulty obtaining primers unique to the region being amplified. None of the somatic events were identified as germline (mutations in both clone and blood) (S6 Table).

LOH events that were either present in protein coding regions, or were in regions with CNVs, were taken for validation by PCR and Sanger sequencing. Only 10 such events were validated due to constraints with the amount of DNA available and difficulties in obtaining primers specific to the genomic regions. The LOH event was considered somatic if it was identified as a heterozygous allele in blood DNA and as a homozygous mutant in the clone (S5 Table).

Structural variants were validated by PCR using primers flanking the novel junction formed and Sanger sequencing the PCR product. The structural change was considered somatic if it was detected in the clone and not detected in the blood DNA (S5 Fig and S4 Table). The plots depicting all somatic changes in the clones were generated using RCircos [77].

### Calculating enrichment and mutation load for mechanistic knowledge-based mutation signature analyses.

The enrichment of UV-induced mutation signatures and mutation load in the clones were calculated similar to [43, 44] We use the term mutation signature in reference to the characteristics of a given mutation including the mutated residue, the altered base and the nucleotides that surround the residue that occur more frequently than expected with random mutations in genomic DNA. The signatures analyzed in this paper are listed in Table 2.

**Table 2:** Mutation signatures analyzed in this study.

Signature motif (abbreviated)	Signature motif (detailed)	Base substitution in a motif (a.k.a. mutation signature)
nCg	5’ [a|t|g|c]C[g] 3’	nCg→nTg
rCg	5’ [a|g]C[g] 3’	rCg→rTg
yCg	5’ [t|c]C[g] 3’	yCg→yTg
yCn	5’ [t|c]C[a|t|g|c] 3’	yCn→yTn
nTt	5’ [a|t|g|c]T[t] 3’	nTt→nCt
rTt	5’ [a|g]T[t] 3’	rTt→rCt
yTt	5’ [t|c]T[t] 3’	yTt→yCt

The mutated residue is capitalized in the center of the motif. In the detailed signature motif, possible nucleotides for a position are show in brackets separated by |.

For UV-signatures, C→T changes in the context of the yCn motif where n is any base and y is pyrimidine (T or C), and T→C changes in the 5’ T in the context of the nTt motif, were analyzed. Example of the calculation for enrichment of mutations within the nTt motif is presented below where, the context is derived from 41-nucleotides region containing the mutated residue in the center.

$${Enrichment}_{\left(nTt\to nCt\right)}=\frac{{mutations}_{\left(nTt\to nCt\right)}\times {\;context}_{\left(t\right)}}{{mutations\;}_{\left(T\to C\right)}\times {context}_{\left(nTt\right)}}$$

For each motif, the reverse complement was also used in the calculations. The use of nucleotide context immediately surrounding the mutation rather than the whole-genome helps to account for localized preference of mutagenesis stemming from small range scanning by mutagenic enzymes such as APOBEC, or the preference of lesion occurrence, lesion repair as well as other factors including epigenomic features influencing mutagenesis within localized genomic regions [78, 79]. These calculations do not exclude any specific genomic region, but rather use the area in each sample, where the mutations actually happened. With large numbers of mutations this approach gives results similar to calculations using whole-genome context [43].

To statistically determine the over-representation of the UV-signature mutations a one-sided Fisher’s exact test was performed. The ratio of the number of C → T (for yCn and nCg) or T →C (for nTt) changes that occurred within or out of their respective motifs were compared to the ratio of C or T bases present within or out of the motifs above. To correct P-values for multiple testing the Benjamini-Hochberg method was used.

The minimum estimate of the number of mutations attributable to UV for both nCy and nTt motifs were calculated only for samples with enrichment > 1 also having corrected q-value for enrichment < 0.05. For samples with enrichment < 1 and for samples with enrichment q-value > 0.05 the minimum estimate of mutation load was assigned the value = 0. For example, minimum mutation load for nTt→nCt was calculated as

$${MutLoad}_{\left(nTt\to nCt\right)}=\frac{{mutations}_{\left(nTt\to nCt\right)}\times ({Enrichment}_{\left(nTt\to nCt\right)}-1)}{{Enrichment}_{\left(nTt\to nCt\right)}}$$

Mutagenesis in CpG leading to nCg→ nTg changes was evaluated in a similar way (Fig 4A and 4B and S11 Table).

Total tandem CC→TT dinucleotide changes were calculated for each sample (Fig 4C and S11 Table). This mutation signature is known to be unique to UV and the probability of random CC→TT changes not by UV is extremely low [50].

In order to assess the contributions of the -1 nucleotide in the nCg and nTt mutation motifs, we also evaluated rCg→rTg, yCg→yTg, rTt→rCt and yTt→yCt mutation signatures as described above.

### Correlation of somatic mutation density with replication timing, chromatin status and transcription

Replication timing for each 1Mb window of the genome was determined by taking the average of the wavelet-smoothed signal. The average replication timing was binned into 5 equal bins and the mean mutation density per Mb was calculated for each bin (Fig 5A, S10 Fig and S12 Table). The total number of DNase I hypersensitive peaks were calculated across 1Mb windows in the human genome. The data was binned into 5 equal bins and mean mutation density was calculated for each bin. BEDtools suite was used to map peaks to 1Mb intervals (Fig 5A, S11 Fig and S12 Table). Variation of UV-induced mutation density with replication timing and DNaseI hypersensitivity levels was also determined for yCn→yTn, nTt→nCt and CC→TT mutations (S12 Fig and S12 Table).

To statistically determine the over-representation of mutations in pyrimidines due to UV in the non-transcribed strand of genes, the number of C→T and T→C mutations in the yCn and nTt contexts and CC→TT mutations were determined for both strands of transcribed genes, and a one sided binomial test was performed expectation that more mutations are present on the non-transcribed strand. To correct P-values for multiple testing the Benjamini-Hochberg method was used. Similar comparison was done for mutations in pyrimidines not conforming to UV-signatures (Fig 5B and S13 Table).

### Code availability

The R-code for analysis of all mutation signatures will be provided on request.

## Supporting Information

S1 Fig Representative image of the fibroblasts isolated from human dermis in this study. (TIF)

S2 Fig Clonality of somatic L1 insertions. Estimated allele frequencies of the two somatic L1 insertions (L1#1 in D1-L-H and L1#2 in D2-R-F clones) were shown in blue and yellow lines, respectively. The histograms of allele frequencies of germline non-reference L1 insertions that were detected from the blood genomes of the two donors and also reported in the literature were shown with (left) and without (right) copy number adjustment. The two major peaks in the histograms represent heterozygous and homozygous germline insertions. The heterozygous germline insertions show slightly higher allele frequencies than 0.5 because some discordant read pairs were counted twice due to additionally derived read pairs from the same DNA fragments. The two somatic L1 insertions were not clonal and likely arose during propagation of cells in culture.
(TIF)

S3 Fig Circos plots depicting all the genetic changes detected in the clones isolated from Donor1. The tracks represent the following features–Track1 (innermost track) = rearrangements detected by Delly (green = deletions, blue = inversions, black = duplications, red = translocations); track2 = deletions as detected by read-depth analyses; track3 = genomic regions with 3N copy number as detected by read-depth analyses; track4 = LOH events; track 5 = SNV positions, red dots = homozygous alleles, black dots = heterozygous alleles and track6 = chromosome ideograms.
(TIF)

S4 Fig Circos plots representing genetic changes in clones from Donor2. The tracks, as numbered from the innermost track, represent: track1 = all rearrangements (green = deletions, blue = inversions, black = duplications, red = translocations); track 2 and track 3 = genomic regions with deletions and amplifications as detected by read-depth analyses, respectively; track 4 = genomic positions for LOH events; track 5 = SNVs (red = homozygous, black = heterozygous); track6 = chromosome ideograms.
(TIF)

S5 Fig Example of validation of somatic structural variants in the clones. The new DNA junction formed was amplified. The structural change (yellow arrow) is present in the clone (C) and absent in blood (B) DNA. If the somatic change is a heterozygous deletion, and is < ~2 kb in length, the full length product can also be amplified (blue arrow) and is expected to be present in both blood and clone DNA. 1 = D1-L-F1 deletion chr5:125315996–125316120; 2 = D1-L-F1 deletion chr18:34125127–34241348; 3 = D1-R-F deletion chr2:24964091–24965103.
(TIF)

S6 Fig Distribution of allele frequencies of the consensus SNVs called by the three mutation callers. The X-axis denotes allele frequencies in 5% increments. The Y-axis represents the percentage of SNVs in each sample with the given allele frequencies. The percentage of mutations with allele frequencies between 45% and 55% and with frequencies > 90%, respectively, is provided.
(TIF)

S7 Fig Validation of shared SNVs between 2 clones. The mutation identical to the 2 clones from the left forearm of Donor1 (chr1:152671788 C→T), and the two mutations present in both the left and right forearms of Donor2 (chr12:433518 C→T and chr14:99090650 A→G) were PCR amplified and Sanger sequenced. In DNA isolated from whole blood, only one peak corresponding to the reference allele is detected. On the other hand, we can detect 2 peaks for the heterozygous reference and mutated alleles in both clones where the mutation is present.
(TIF)

S8 Fig Distribution of allele frequencies of the SNVs detected in the bulk exomes. X-axis denotes allele frequency in increments of 5%, and the Y-axis represents the number of SNVs at the given allele frequency.
(TIF)

S9 Fig Analysis of the nucleotide preference at the +1 position in the nCg→nTg and nTt→nCt mutation signatures. (A) Comparison of the fold enrichment and minimum mutation loads of the rCg →rTg (blue bars) and yCg→yTg (yellow bars) mutation signatures (r is any purine and y is any pyrimidine). (B) Comparison of the fold enrichment and minimum mutation loads of the rTt→rCt (pink bars) and yTt→yCt (orange bars) mutation signatures. Black line depicts enrichment = 1.
(TIF)

S10 Fig Correlation of mutation density from all clones with replication timing. The bins on the X-axis denote the wavelet-smoothed signal for replication timing per 1Mb genome window divided into 5 equal bins. All samples have positive slopes indicating that mutation density increases in later replicating regions.
(TIF)

S11 Fig Correlation of mutation density in all clones with DNase I hypersensitivity. The bins on the X axis were obtained by calculating the number of DNase I hypersensitive sites per 1 Mb genome and dividing them into 5 bins. Almost all samples demonstrate higher mutation density in regions of the genome with closed chromatin as compared to open chromatin.
(TIF)

S12 Fig Correlation of UV-induced mutation density from forearm samples with replication timing and DNase I hypersensitivity. The average mutation density in each bin for all forearm samples is plotted. The bins on the X-axis were obtained by dividing wavelet-smoothed signal for replication timing and total number of DNase I hypersensitive peaks, per 1Mb genome window, into 5 equal bins. Increasing bin values denote later replication timing, and higher heterochromatin levels (transition from open to closed chromatin).
(TIF)

S1 Table Coverage statistics for clones WGS and bulk samples WES. (XLSX)

S2 Table L1 retrotransposition events in clones. (XLSX)

S3 Table Copy number changes detected in clones. (XLSX)

S4 Table Somatic structural variants detected in the clones. (XLSX)

S5 Table Loss of heterozygosity of SNVs detected in the fibroblasts. (XLSX)

S6 Table List of somatic SNVs present in the exons of the fibroblast clones analyzed. (XLSX)

S7 Table Distribution of allele frequencies for the consensus SNVs called by the triple caller. (XLSX)

S8 Table Distribution of allele frequencies in the exomes of the bulk samples sequenced. (XLSX)

S9 Table Comparison of the observed number of SNVs detected in the bulk cells with the expected number from the number of changes detected in the clones. (XLSX)

S10 Table Somatic mutation rates in all clones. (XLSX)

S11 Table Motif-specific mutation enrichment and minimum mutation loads. (XLSX)

S12 Table Distribution of mutation load with replication timing and DnaseI hypersensitivity levels in the genome. (XLSX)

S13 Table Distribution of UV-induced and other mutations on the non-transcribed and transcribed strands of transcribed regions. (XLSX)
