# Supplementary figures and images for: The Impact of Environmental and Endogenous Damage on Somatic Mutation Load in Human Skin Fibroblasts
Source: PLoS Genet. 2016 Oct 27;12(10):e1006385. doi: 10.1371/journal.pgen.1006385 (PMC5082821; doi:10.1371/journal.pgen.1006385)

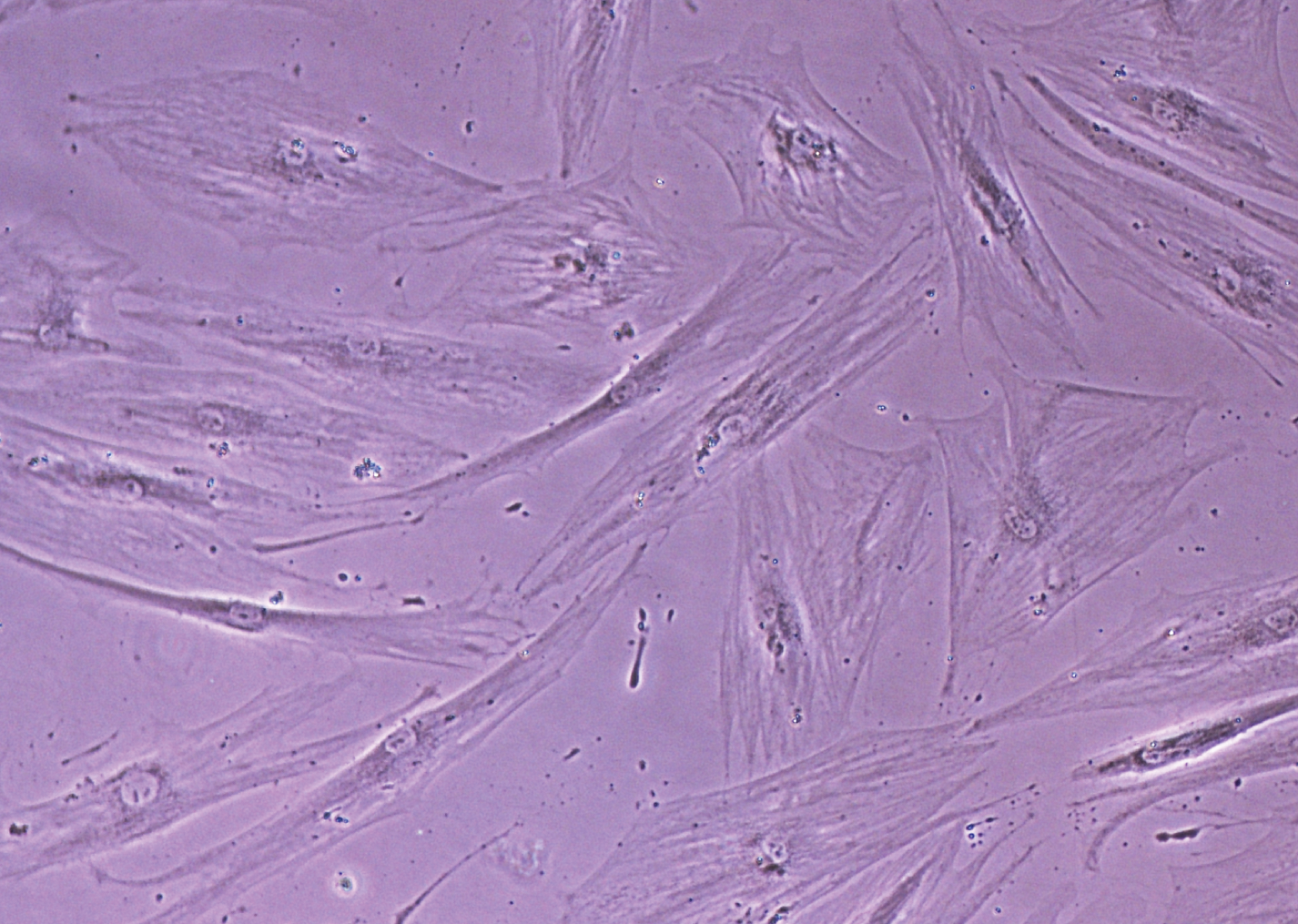

Supplement: S1 Fig — (TIF) [file pgen.1006385.s001.tif]

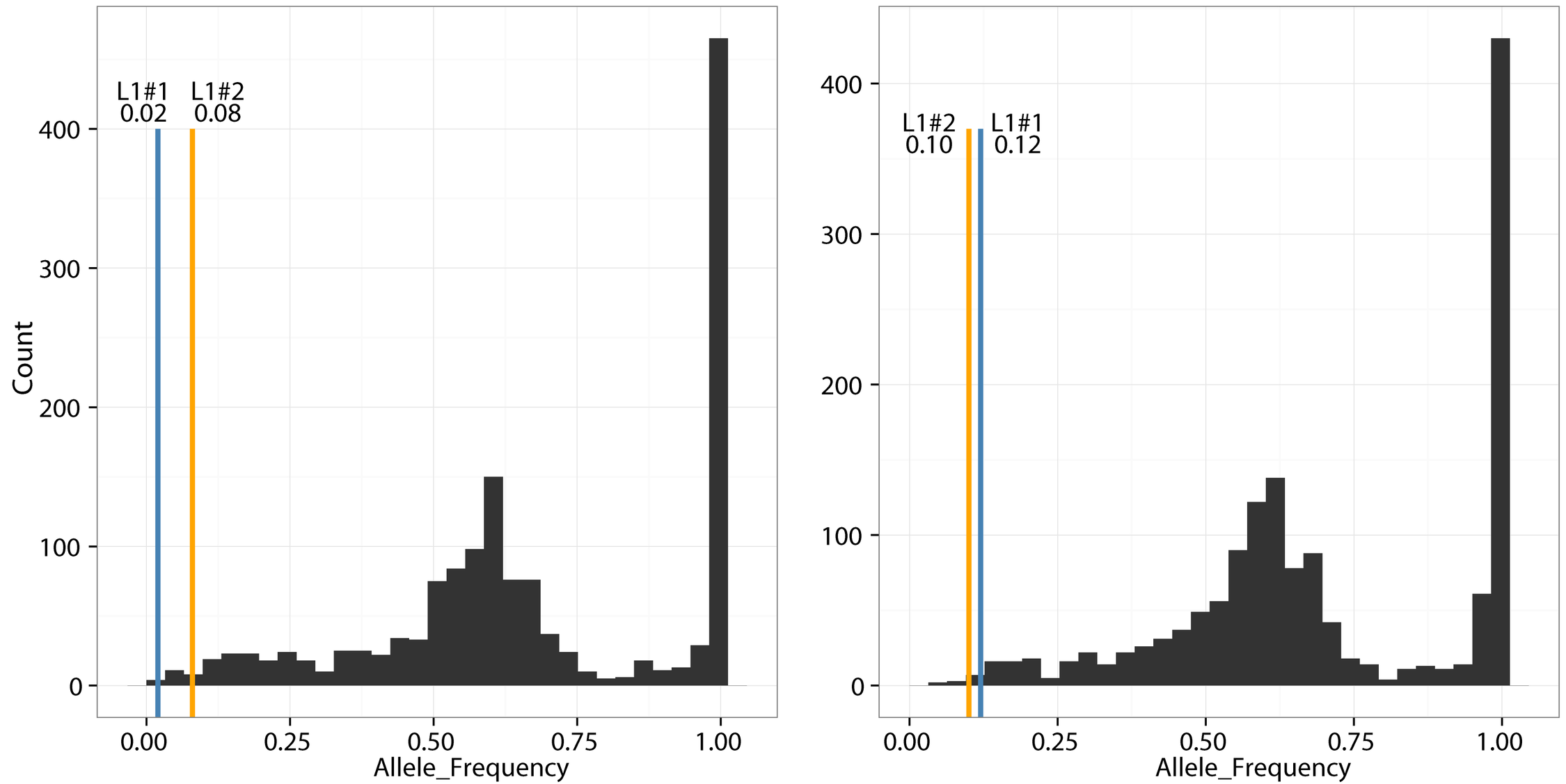

Supplement: S2 Fig — Estimated allele frequencies of the two somatic L1 insertions (L1#1 in D1-L-H and L1#2 in D2-R-F clones) were shown in blue and yellow lines, respectively. The histograms of allele frequencies of germline non-reference L1 insertions that were detected from the blood genomes of the two donors and also reported in the literature were shown with (left) and without (right) copy number adjustment. The two major peaks in the histograms represent heterozygous and homozygous germline insertions. The heterozygous germline insertions show slightly higher allele frequencies than 0.5 because some discordant read pairs were counted twice due to additionally derived read pairs from the same DNA fragments. The two somatic L1 insertions were not clonal and likely arose during propagation of cells in culture. (TIF) [file pgen.1006385.s002.tif]

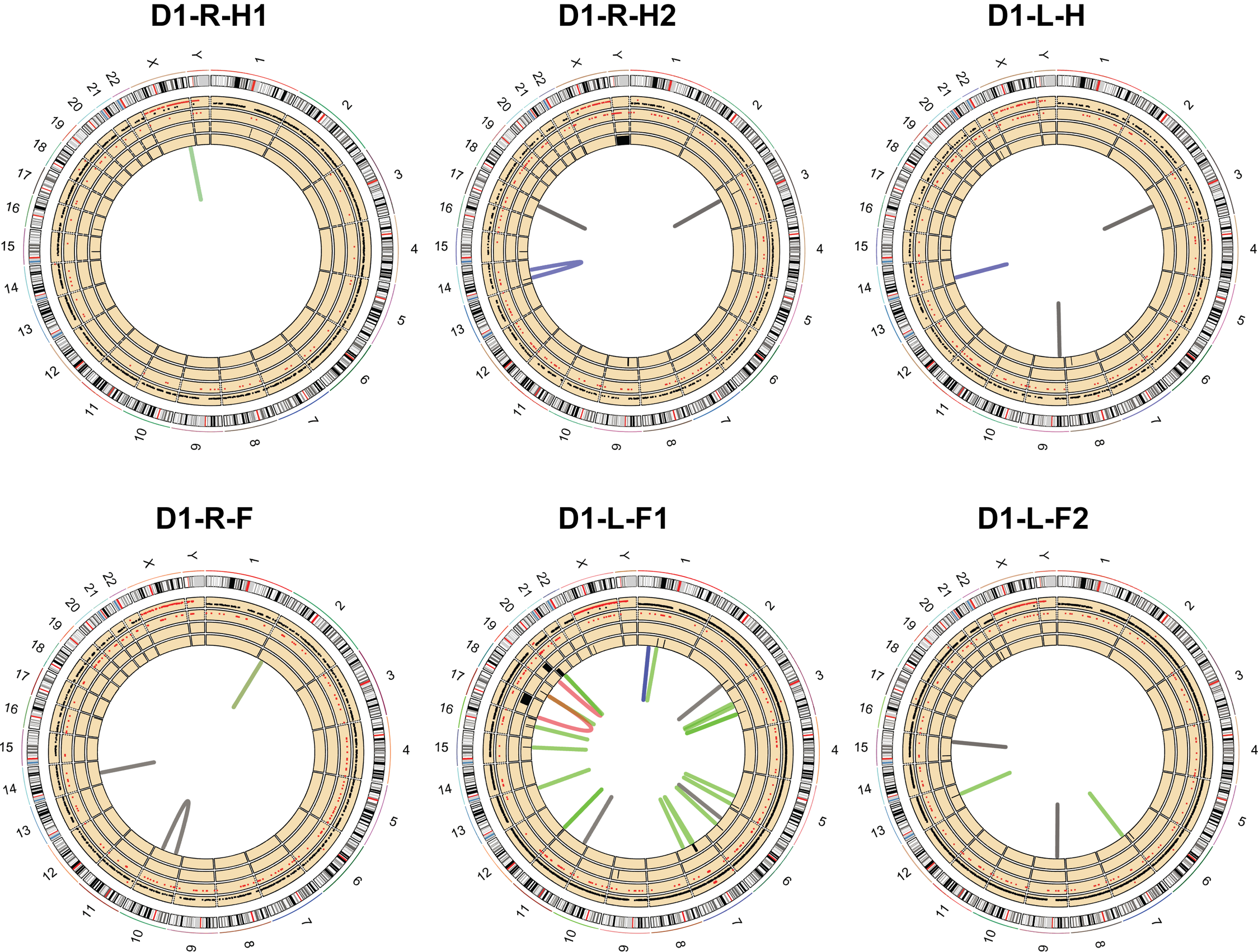

Supplement: S3 Fig — The tracks represent the following features–Track1 (innermost track) = rearrangements detected by Delly (green = deletions, blue = inversions, black = duplications, red = translocations); track2 = deletions as detected by read-depth analyses; track3 = genomic regions with 3N copy number as detected by read-depth analyses; track4 = LOH events; track 5 = SNV positions, red dots = homozygous alleles, black dots = heterozygous alleles and track6 = chromosome ideograms. (TIF) [file pgen.1006385.s003.tif]

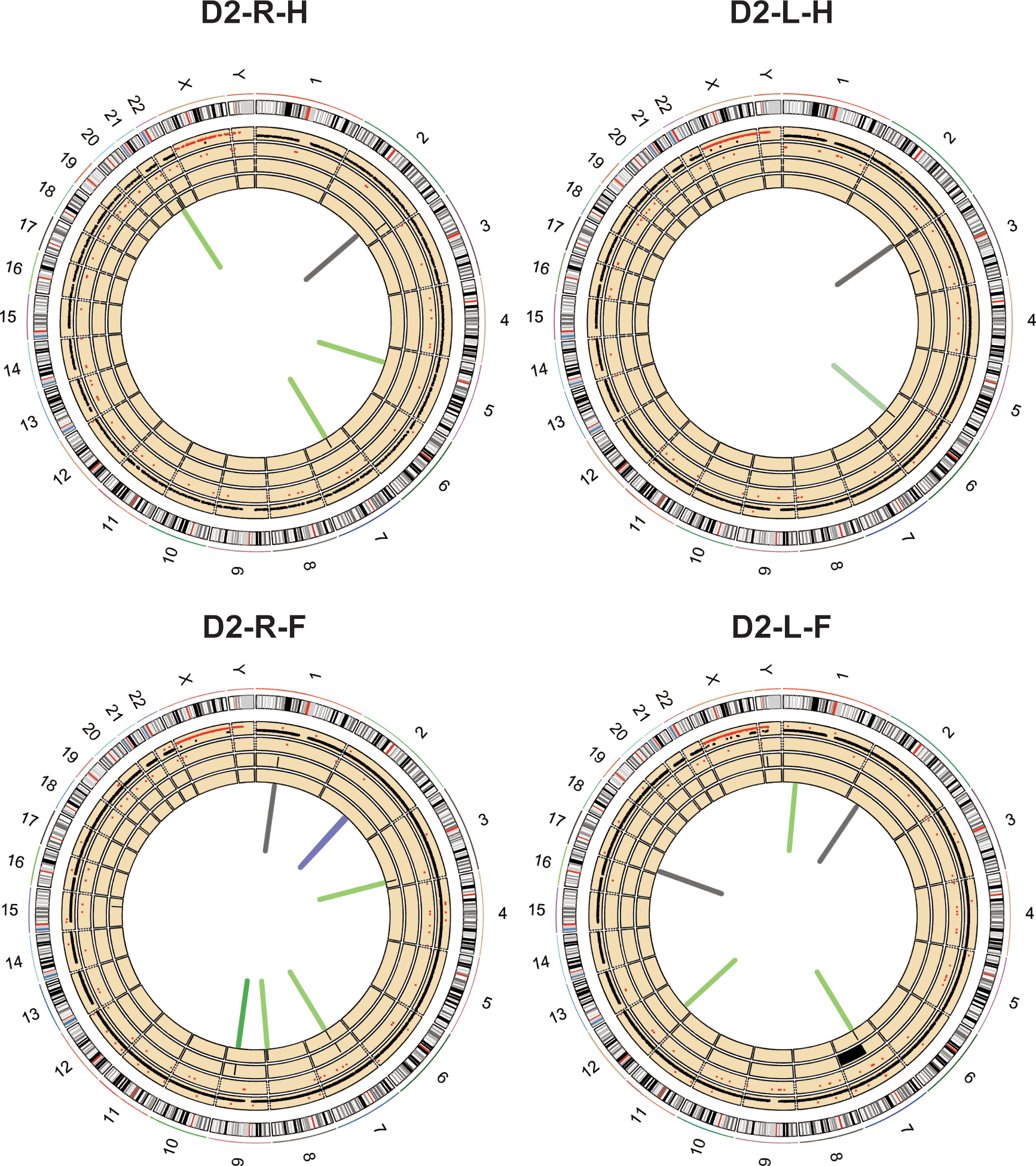

Supplement: S4 Fig — The tracks, as numbered from the innermost track, represent: track1 = all rearrangements (green = deletions, blue = inversions, black = duplications, red = translocations); track 2 and track 3 = genomic regions with deletions and amplifications as detected by read-depth analyses, respectively; track 4 = genomic positions for LOH events; track 5 = SNVs (red = homozygous, black = heterozygous); track6 = chromosome ideograms. (TIF) [file pgen.1006385.s004.tif]

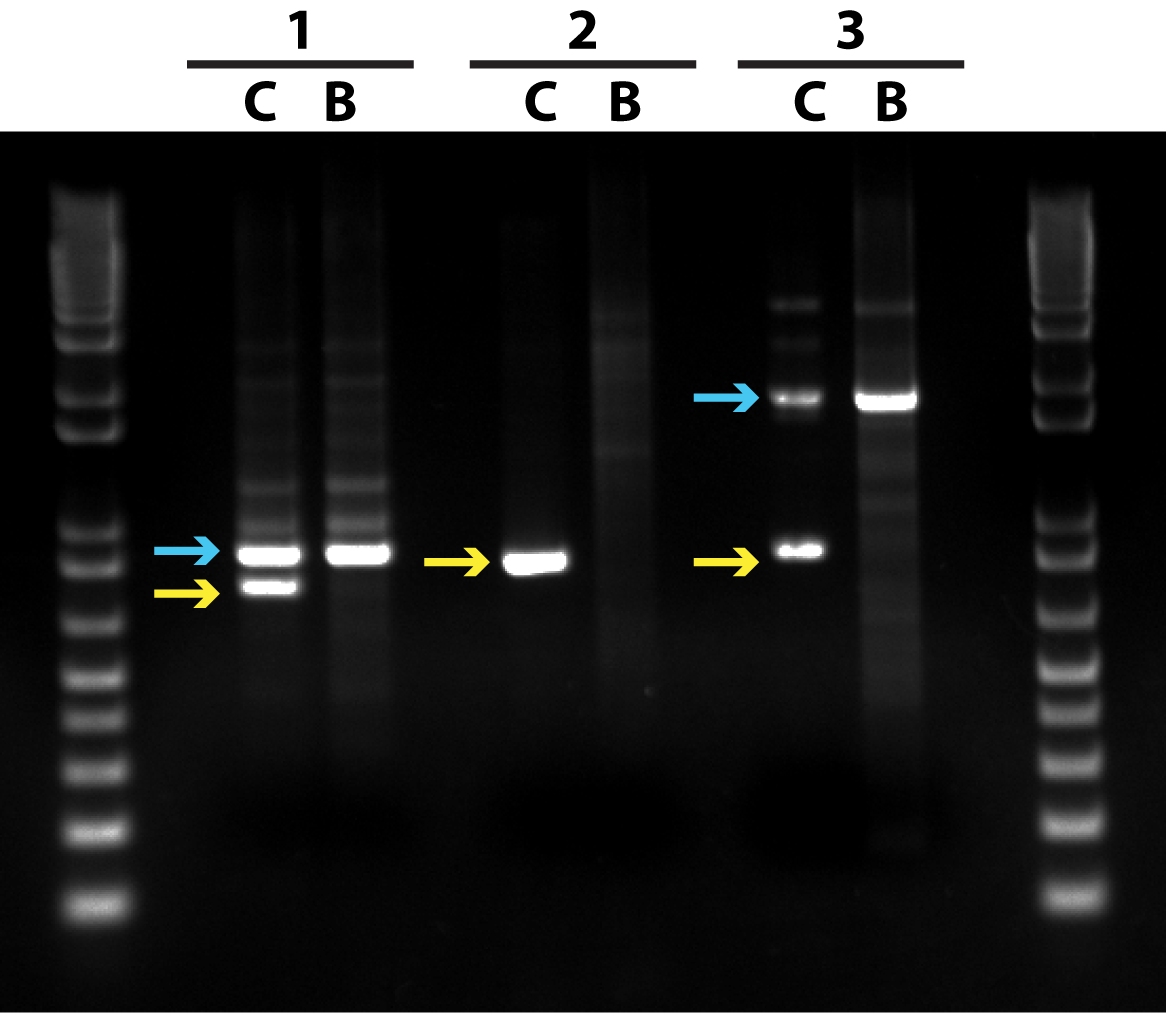

Supplement: S5 Fig — The new DNA junction formed was amplified. The structural change (yellow arrow) is present in the clone (C) and absent in blood (B) DNA. If the somatic change is a heterozygous deletion, and is < ~2 kb in length, the full length product can also be amplified (blue arrow) and is expected to be present in both blood and clone DNA. 1 = D1-L-F1 deletion chr5:125315996–125316120; 2 = D1-L-F1 deletion chr18:34125127–34241348; 3 = D1-R-F deletion chr2:24964091–24965103. (TIF) [file pgen.1006385.s005.tif]

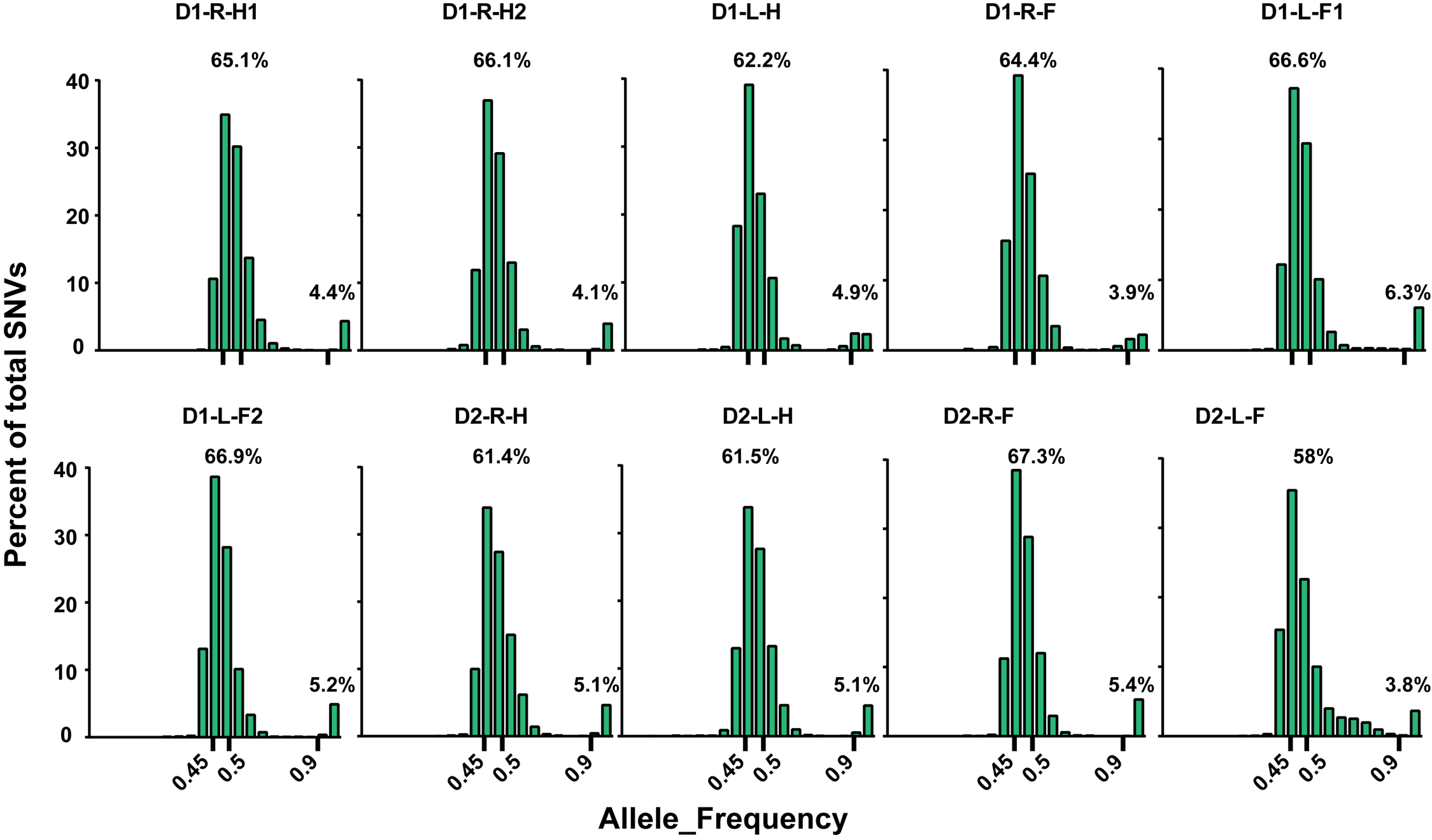

Supplement: S6 Fig — The X-axis denotes allele frequencies in 5% increments. The Y-axis represents the percentage of SNVs in each sample with the given allele frequencies. The percentage of mutations with allele frequencies between 45% and 55% and with frequencies > 90%, respectively, is provided. (TIF) [file pgen.1006385.s006.tif]

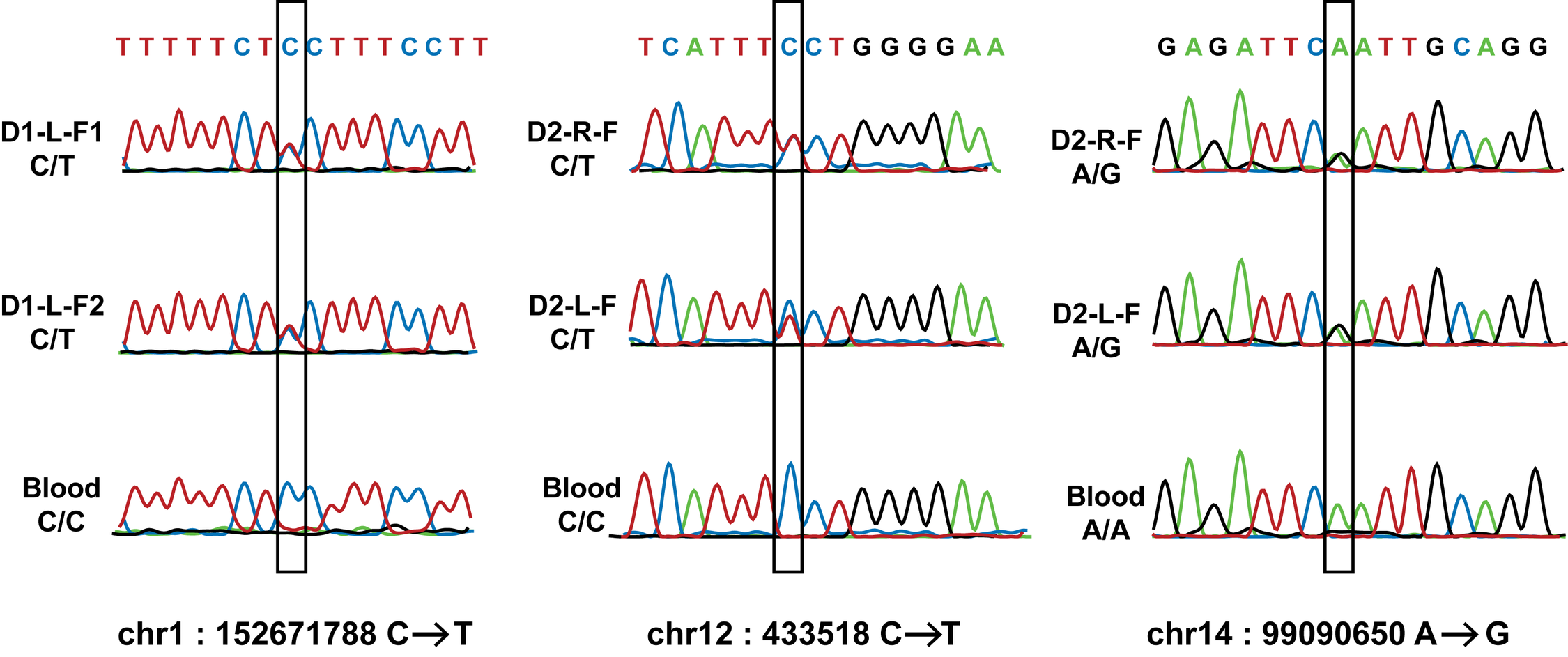

Supplement: S7 Fig — The mutation identical to the 2 clones from the left forearm of Donor1 (chr1:152671788 C→T), and the two mutations present in both the left and right forearms of Donor2 (chr12:433518 C→T and chr14:99090650 A→G) were PCR amplified and Sanger sequenced. In DNA isolated from whole blood, only one peak corresponding to the reference allele is detected. On the other hand, we can detect 2 peaks for the heterozygous reference and mutated alleles in both clones where the mutation is present. (TIF) [file pgen.1006385.s007.tif]

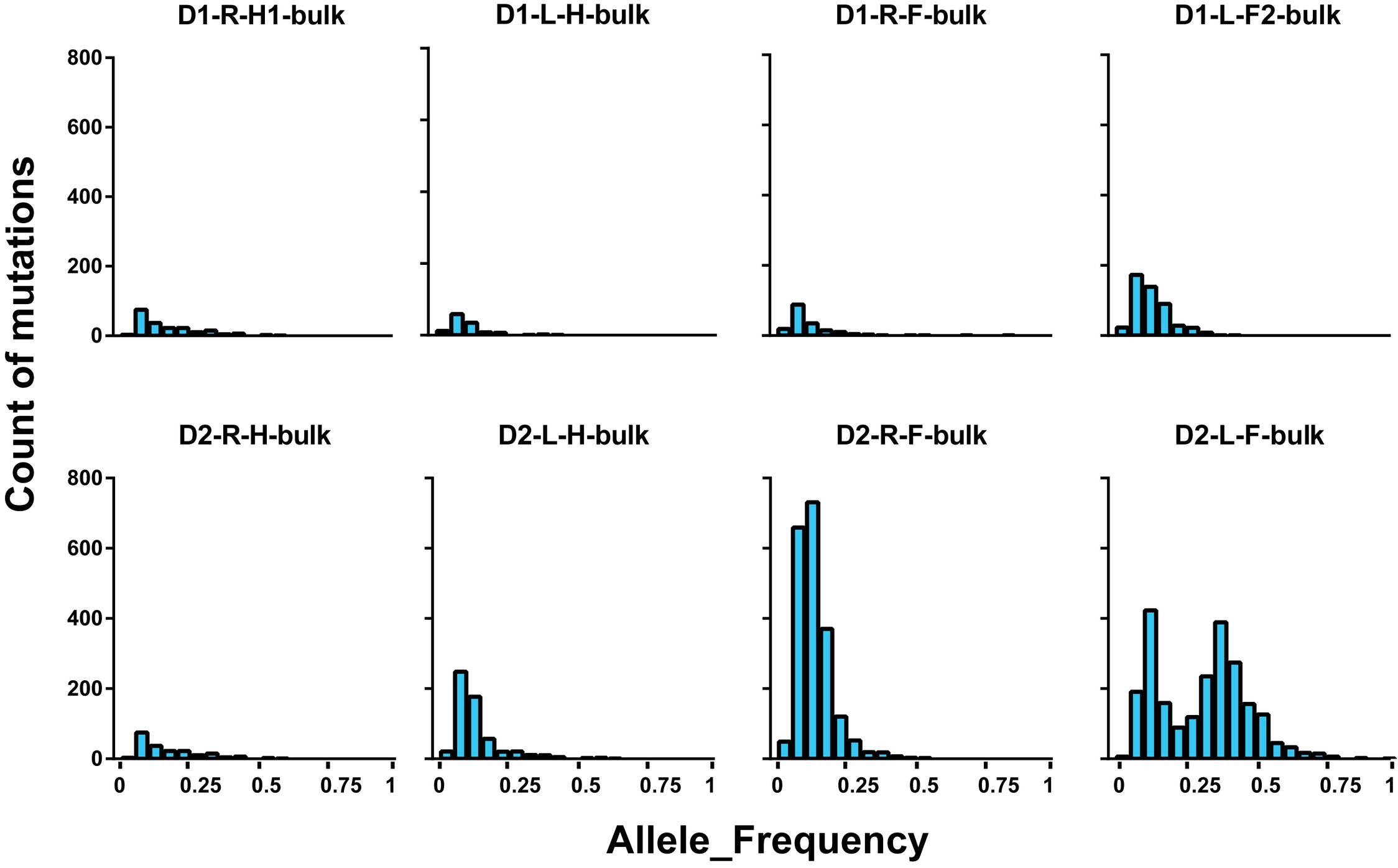

Supplement: S8 Fig — X-axis denotes allele frequency in increments of 5%, and the Y-axis represents the number of SNVs at the given allele frequency. (TIF) [file pgen.1006385.s008.tif]

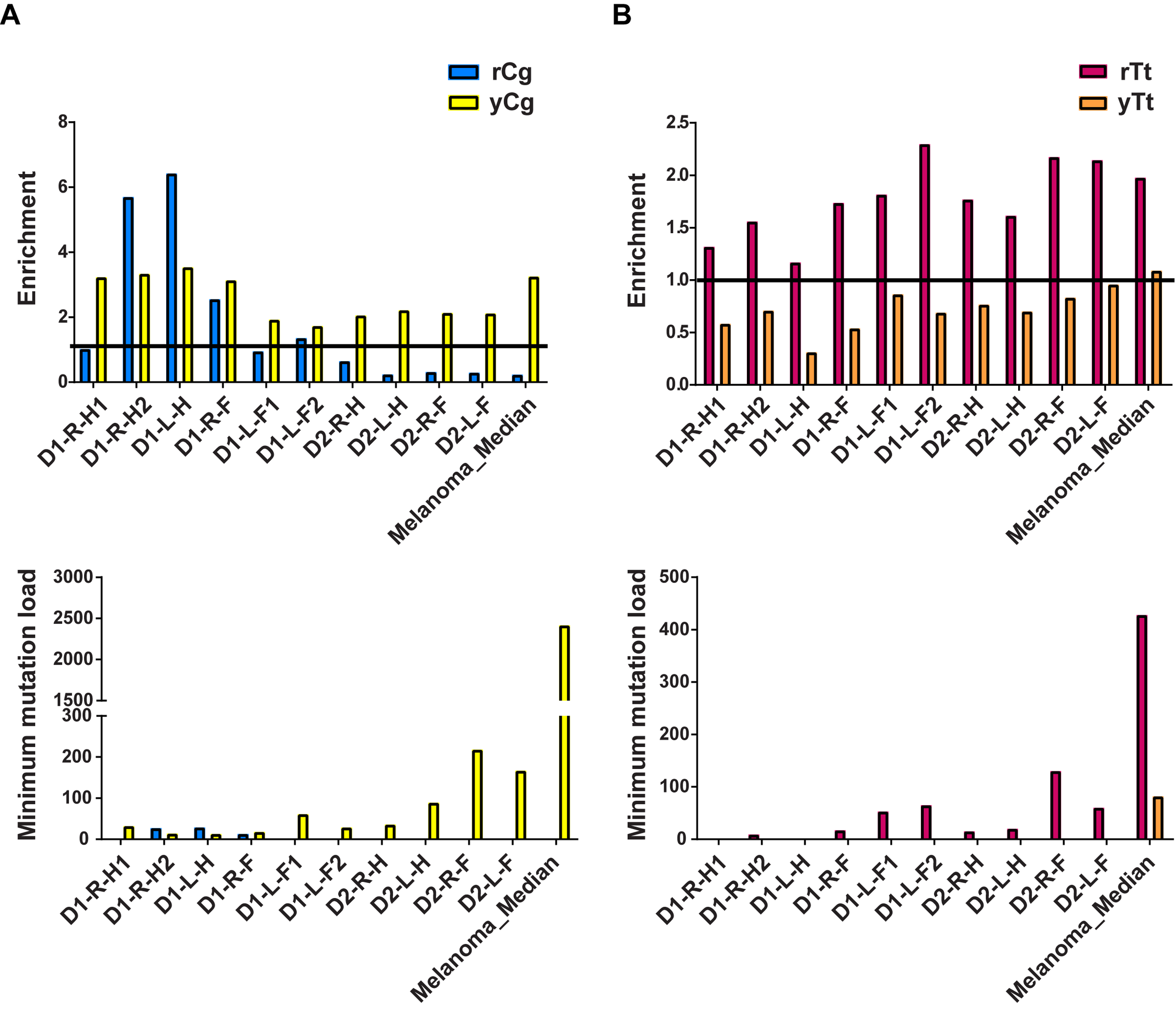

Supplement: S9 Fig — (A) Comparison of the fold enrichment and minimum mutation loads of the rCg →rTg (blue bars) and yCg→yTg (yellow bars) mutation signatures (r is any purine and y is any pyrimidine). (B) Comparison of the fold enrichment and minimum mutation loads of the rTt→rCt (pink bars) and yTt→yCt (orange bars) mutation signatures. Black line depicts enrichment = 1. (TIF) [file pgen.1006385.s009.tif]

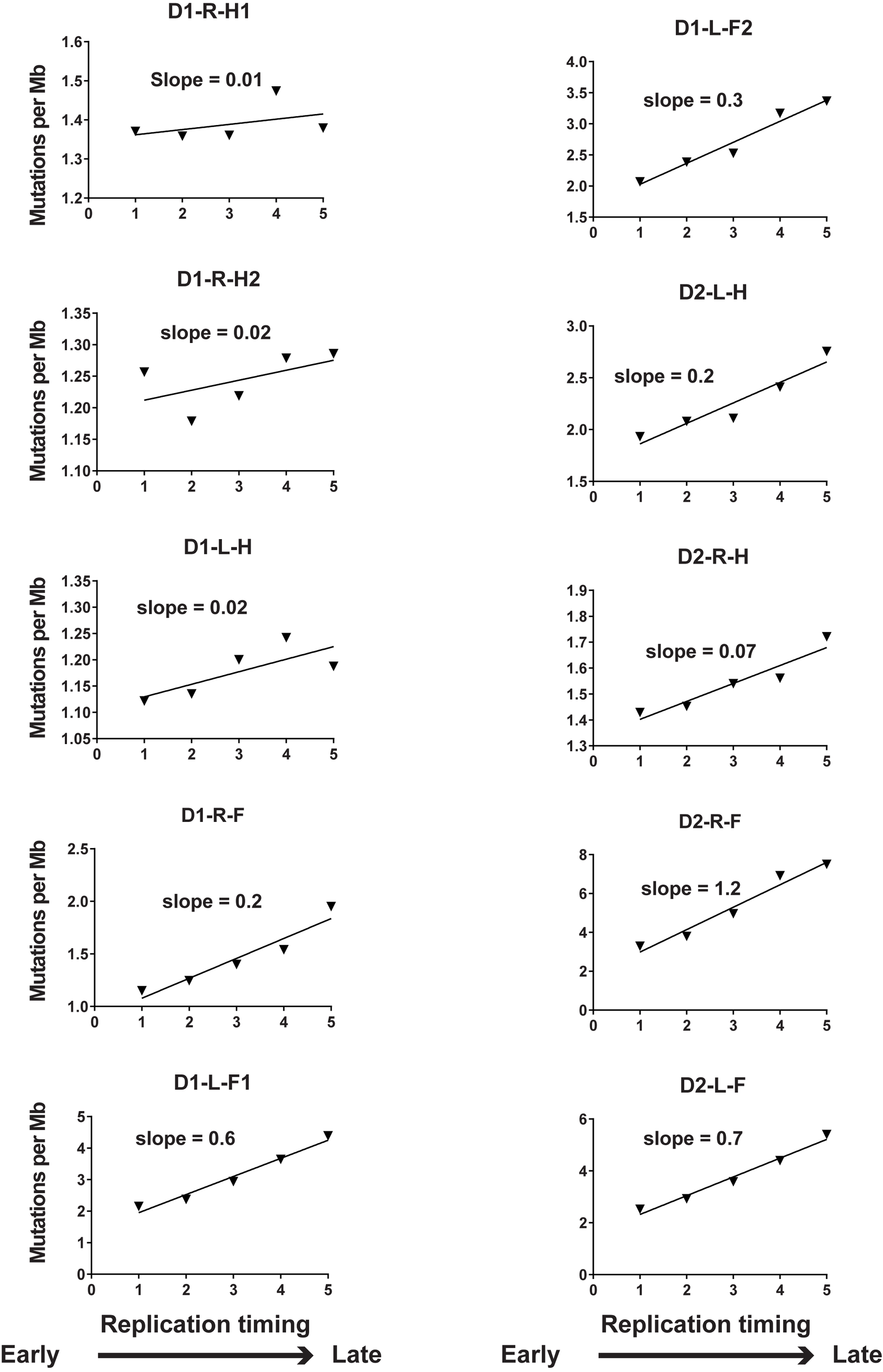

Supplement: S10 Fig — The bins on the X-axis denote the wavelet-smoothed signal for replication timing per 1Mb genome window divided into 5 equal bins. All samples have positive slopes indicating that mutation density increases in later replicating regions. (TIF) [file pgen.1006385.s010.tif]

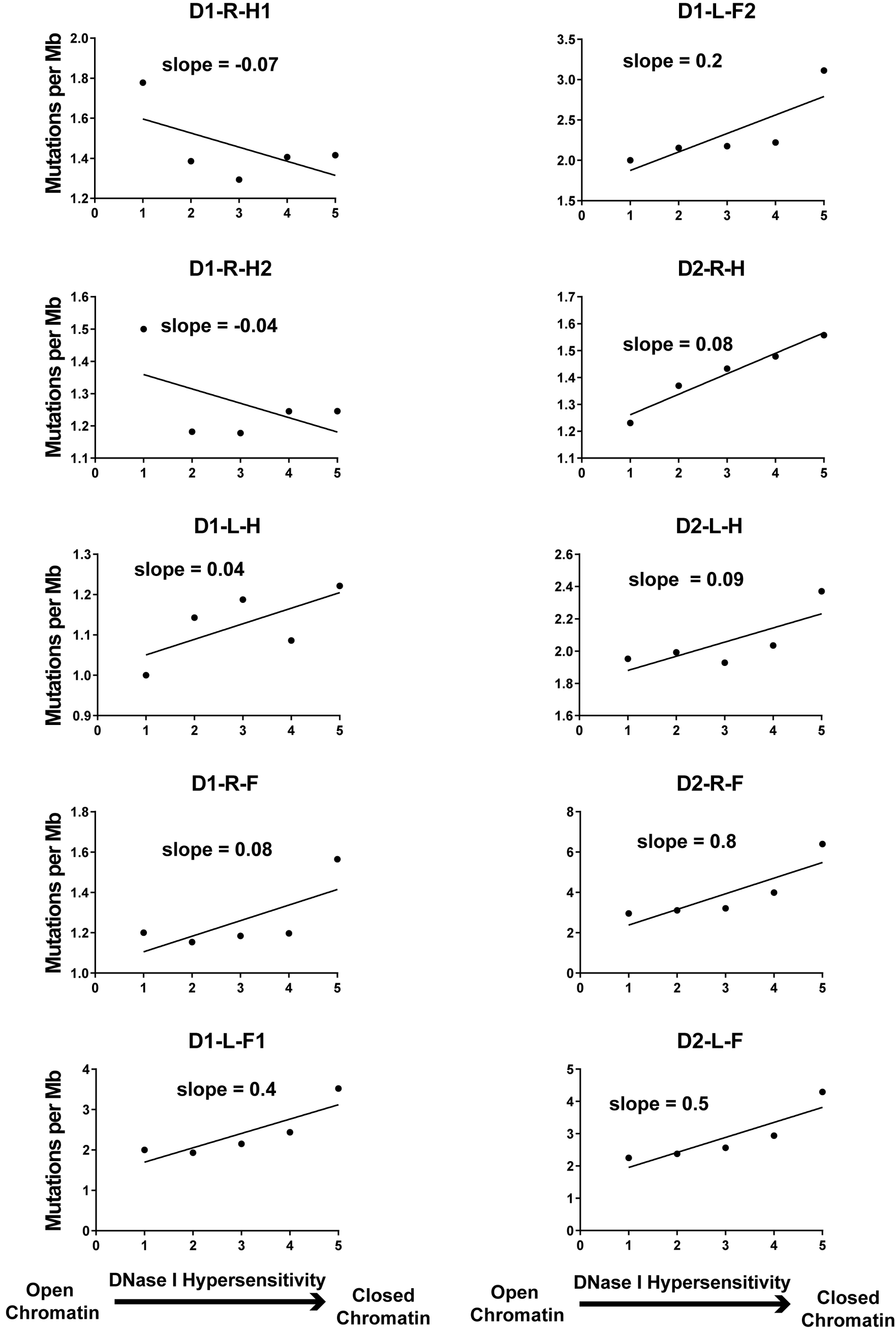

Supplement: S11 Fig — The bins on the X axis were obtained by calculating the number of DNase I hypersensitive sites per 1 Mb genome and dividing them into 5 bins. Almost all samples demonstrate higher mutation density in regions of the genome with closed chromatin as compared to open chromatin. (TIF) [file pgen.1006385.s011.tif]

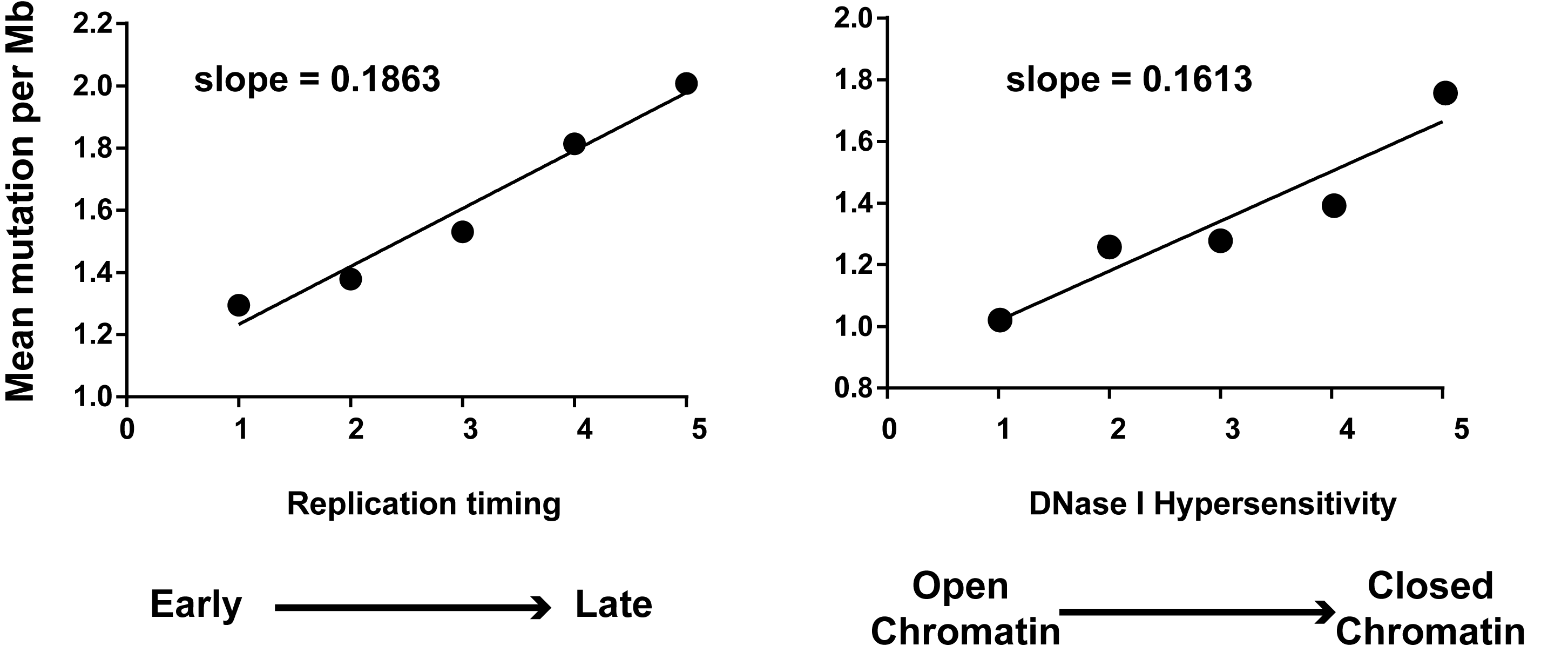

Supplement: S12 Fig — The average mutation density in each bin for all forearm samples is plotted. The bins on the X-axis were obtained by dividing wavelet-smoothed signal for replication timing and total number of DNase I hypersensitive peaks, per 1Mb genome window, into 5 equal bins. Increasing bin values denote later replication timing, and higher heterochromatin levels (transition from open to closed chromatin). (TIF) [file pgen.1006385.s012.tif]
